# Integrated transcriptomic analysis identifies liver aging-driven fibrosis signatures and reveals therapeutic strategies based on medicine-food homology

**DOI:** 10.1038/s41538-026-00904-6

**Published:** 2026-05-26

**Authors:** Yingqi Xu, Maohao Li, Lun Zhu, Yawen Luo, Wanlu Sheng, Panlei Jia, Rina Suo, Lidao Bao

**Affiliations:** 1 College of Bioinformatics Science and Technology, Harbin Medical University, Harbin, China; 2 Hohhot Mongolian Medicine of Traditional Chinese Medicine Hospital, Hohhot, China; 3 Department of Cell Fate and Diseases, Jilin Provincial Key Laboratory of Women’s Reproductive Health, Jilin Provincial Clinical Research Center for Birth Defect and Rare Disease, The First Hospital of Jilin University, Changchun, China; 4 College of Pharmacy, Inner Mongolia Medical University, Hohhot, China; 5 Hohhot First Hospital, Hohhot, China; 6 Inner Mongolia Autonomous Region Rehabilitation Center for Children with Disabilities, Hohhot, China

**Keywords:** Computational biology and bioinformatics, Diseases, Drug discovery

## Abstract

Despite its strong regenerative capacity, liver aging paradoxically increases susceptibility to fibrosis and metabolic dysfunction-associated steatotic liver disease through dysregulated inflammation, senescence-associated secretory phenotypes, and immune-metabolic crosstalk. To systematically characterize these processes, we integrated longitudinal transcriptomics, single-cell RNA sequencing, and machine-learning approaches. We identified 252 aging-associated genes and developed an Aging Gene Score (AGS) to quantify senescence burden across hepatic cell populations. Single-cell analysis revealed macrophages as key drivers of fibrosis progression, with high-AGS myeloid subsets markedly expanded in cirrhotic livers. Using combined Boruta and LASSO algorithms, we established a five-gene biomarker panel (*EFEMP1*, *LUM*, *DKK3*, *GPRC5B*, *NCAM2*) that accurately predicts advanced fibrosis (AUC > 0.77). Furthermore, a network pharmacology framework was applied to screen medicine-food homology (MFH) herbs, identifying *Fagopyrum dibotrys* (Jinqiaomai) and *Astragalus membranaceus* (Huangqi) as top candidates. Molecular docking demonstrated strong binding between the bioactive compound MOL000098 and the fibrosis-related target *COL3A1*. Functional assays showed that Jinqiaomai-containing serum alleviates oxidative stress, improves HepG2 cell viability, reduces ALT and AST levels, and suppresses macrophage lipid accumulation, accompanied by reduced expression of inflammatory and fibrosis-related markers. Collectively, our findings highlight macrophage-centered mechanisms linking liver aging and fibrosis and suggest MFH-derived compounds as promising anti-aging and anti-fibrotic dietary interventions.

## Introduction

Aging is a gradual and irreversible degenerative process of cells, tissues, and organs that increases the risk of functional decline and mortality^1^. The liver, with its unique regenerative capacity, has garnered significant attention during aging. However, despite this regenerative ability, liver aging markedly elevates the risk of diseases such as metabolic dysfunction-associated steatotic liver disease, cirrhosis, and liver cancer^2^. Additionally, as the core organ for metabolic regulation, liver aging not only compromises hepatic health but also influences the function of other organs, including the heart and nervous system, through the secretion of hepatokines^3^.

The relationship between liver aging and fibrosis is complex, involving multiple cell types and molecular mechanisms. As an individual age, the liver undergoes structural and functional changes, such as reduced blood flow, diminished regenerative capacity, and exacerbated immune inflammation and fibrosis^4^. Aging hepatocytes, for example, secrete pro-inflammatory factors (senescence-associated secretory phenotype, SASP) that activate surrounding cells, thereby promoting the activation of hepatic stellate cells (HSCs) and subsequent fibrosis^5^. Conversely, the senescence of HSCs may inhibit fibrosis progression by inducing cell cycle arrest and enhancing extracellular matrix degradation^6^. In addition, senescence in other hepatic cell types—such as macrophages, endothelial cells, and natural killer (NK) cells—has been reported to exert context-dependent effects on inflammation and fibrogenesis, underscoring the cellular heterogeneity underlying age-associated liver pathology^7–9^.

Among these cell populations, hepatic macrophages have been extensively implicated as key regulators of liver fibrosis through their ability to orchestrate inflammatory signaling, activate HSCs, and modulate extracellular matrix remodeling via pathways such as TGF-β, NF-κB, and chemokine-mediated crosstalk^10,11^. Most existing studies have focused on macrophage activation states or polarization phenotypes in fibrotic or inflammatory settings, yielding important insights into macrophage-driven fibrogenesis^12,13^. However, these mechanisms have largely been examined in static pathological contexts and have rarely incorporated the temporal dimension of chronological aging. Consequently, it remains unclear how aging-associated transcriptional programs emerge and accumulate during macrophage differentiation, or how these programs progressively reprogram macrophage immune and fibrogenic phenotypes along the aging-fibrosis continuum.

Concurrently, advances in the molecular understanding of liver aging have highlighted senescence-targeted interventions as promising therapeutic strategies. A proteolysis-targeting chimera (PROTAC) targeting BCL-xL/BCL-2, for instance, can selectively eliminate senescent liver cells, thereby delaying the progression of metabolic liver diseases and hepatocellular carcinoma^14^. In studies of alcoholic liver disease, the use of senolytic compound ABT263, which clears senescent cells, has been shown to improve hepatocyte function, modulate lipid metabolism, and reduce inflammation^15^. Furthermore, targeting key signaling pathways, such as ferroptosis, antioxidation, and TGF-β signaling, offers new strategies for mitigating liver aging and its systemic effects^16,17^.

The concept of medicine food homology (MFH) therapy is also emerging as an important approach in preventing and treating liver aging and its related diseases. Traditional Chinese medicine holds that many foods and herbs not only provide nutritional benefits but also contain bioactive compounds with antioxidant, anti-inflammatory, and metabolic regulatory properties. Recent studies have demonstrated that natural compounds such as quercetin, curcumin, and resveratrol exhibit significant potential in reducing hepatic lipid accumulation, alleviating oxidative stress, and inhibiting fibrosis progression^18^. By integrating traditional formulations with modern pharmacology, this approach may offer a safe, low-toxicity, multi-component intervention strategy.

Therefore, this study aims to systematically identify liver-specific aging genes and delineate a macrophage-centered aging-fibrosis crosstalk framework by integrating bulk transcriptomics, single-cell analyses, and machine learning approaches. By establishing a robust, time-resolved aging gene signature from longitudinal liver transcriptomic data and projecting this signature onto single-cell and bulk datasets, we quantify aging-associated transcriptional states at cellular resolution and uncover a progressive accumulation of high-aging-score states along macrophage differentiation trajectories. This integrative strategy enables us to link aging-driven macrophage transcriptional rewiring to pro-fibrotic immune signaling and clinical fibrosis severity. Through comprehensive molecular and cellular analyses, we further identify aging-related biomarkers and potential therapeutic targets with predictive value for fibrosis progression. Collectively, our findings provide new insights into how chronological aging reshapes macrophage-stromal interactions to drive fibrotic remodeling, extending beyond existing macrophage-fibrosis paradigms and offering a conceptual framework to support precision prevention and treatment of aging-related liver diseases and the improvement of health span in the elderly.

## Results

### Identification of genes associated with chronological aging in the liver

**Fig. 1:**
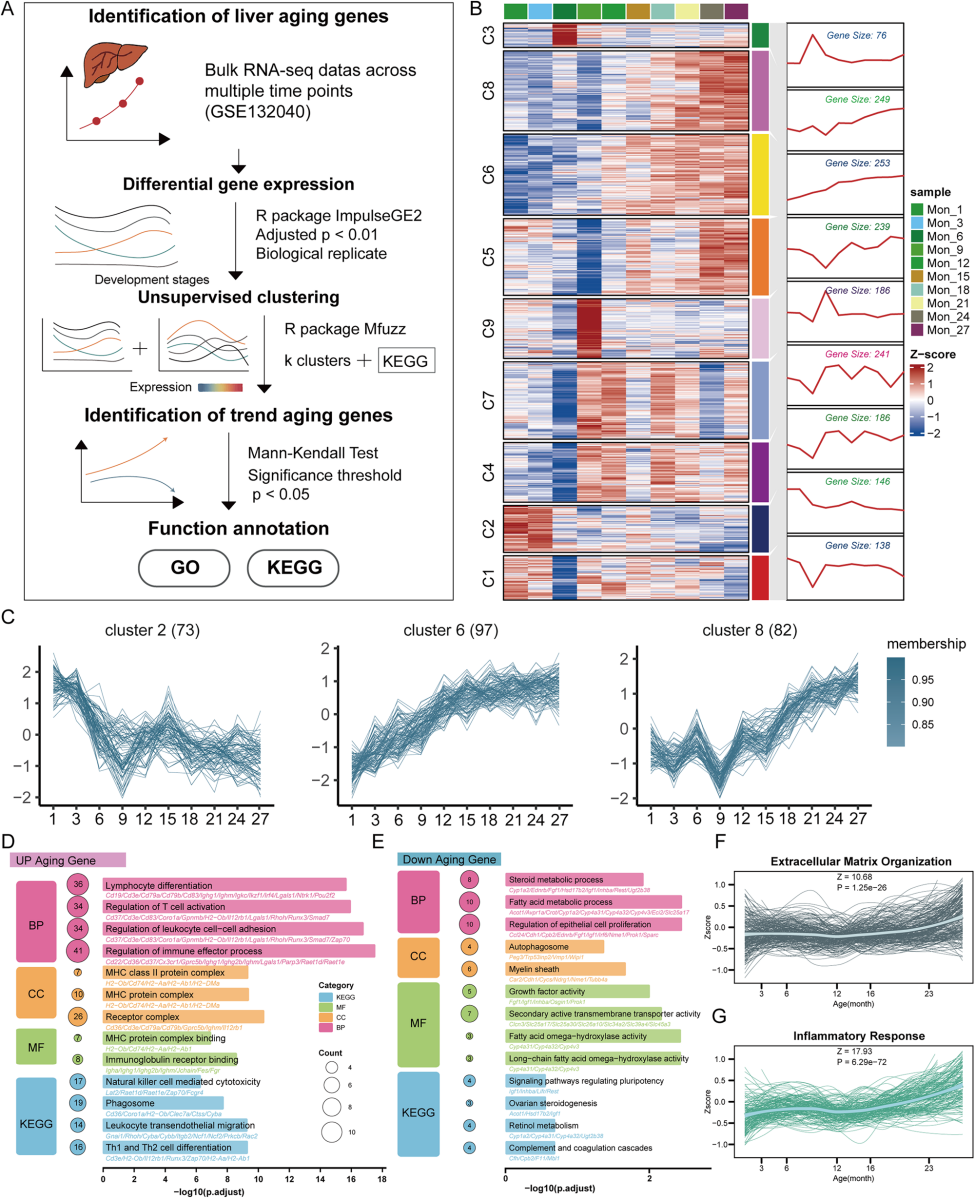
Identification and functional characterization of liver-specific aging-related genes. **A** Schematic diagram illustrating the analytical workflow for identifying liver-specific aging-related genes, including data source, differential expression analysis across age groups, and downstream clustering and functional enrichment analyses. **B** Temporal expression dynamics of differentially expressed genes (DEGs) during aging across mouse liver samples from 1, 3, 6, 9, 12, 15, 18, 21, 24, and 27 months (GSE132040). DEGs were grouped into nine distinct expression clusters based on their longitudinal transcriptional patterns across age stages. **C** Mfuzz soft clustering of liver-specific aging genes according to their dynamic transcriptional trajectories. Color intensity reflects the degree of gene membership within each cluster, with darker colors indicating higher cluster membership values. **D**, **E** Functional enrichment analyses of liver-specific aging genes. Gene Ontology (GO) and KEGG pathway enrichment analyses were performed for upregulated (**D**) and downregulated (**E**) gene sets, with GO terms categorized into biological process (BP), cellular component (CC), and molecular function (MF). **F, G** Age-dependent expression trends of genes derived from Gene Ontology (GO) gene sets for extracellular matrix organization (GO:0031012) (**F**) and inflammatory response (GO:0006954) (**G**), modeled using LOESS regression. These analyses reflect pathway-level transcriptional dynamics across aging.

To systematically identify genes exhibiting significant dynamic changes during liver aging, we employed an integrated approach combining time-series differential expression analysis with gene expression pattern clustering (Fig. 1A). Initially, time-series RNA-seq data from mouse liver tissues collected across 10 distinct age groups (1, 3, 6, 9, 12, 15, 18, 21, 24, and 27 months; GSE132040) were analyzed using the ImpulseDE2 R package. Applying a stringent significance threshold (adjusted *p* < 0.01), we identified 1714 differentially expressed genes (DEGs). Subsequently, these DEGs were subjected to soft clustering using the Mfuzz algorithm, which partitioned the genes into nine distinct temporal expression clusters (Fig. 1B). To pinpoint clusters exhibiting statistically significant monotonic trends over time, we applied the Mann-Kendall (MK) trend test. This analysis revealed that genes within Cluster 2 (C2) displayed a consistent downward trajectory, whereas those in Cluster 6 (C6) and Cluster 8 (C8) showed progressively increasing expression patterns over time. To enhance the reliability of the identified gene sets, we imposed a stringent membership cutoff (membership score > 0.8), yielding 73, 97, and 82 high-confidence genes from clusters C2, C6, and C8, respectively. Integrating these sets resulted in a final collection of 252 aging genes associated with liver aging (Fig. 1C).

To elucidate the potential molecular mechanisms underlying liver aging, we conducted Gene Ontology (GO) and Kyoto Encyclopedia of Genes and Genomes (KEGG) enrichment analyses on the 252 aging trend genes identified (Fig. 1D, E). Functional enrichment revealed that upregulated aging trend genes were significantly associated with immune regulatory processes and major histocompatibility complex (MHC)-related pathways. In contrast, downregulated genes were predominantly enriched in functions related to growth factor activity. Given that liver fibrosis represents a hallmark of age-related liver pathology, we further retrieved gene sets corresponding to inflammatory response (GO:0006954) and extracellular matrix organization (GO:0031012) from the Gene Ontology (GO) database, both of which are closely linked to the development of liver fibrosis during aging. Using locally weighted scatterplot smoothing (LOESS) regression, we modeled the age-dependent expression trajectories of genes within these pathways (Fig. 1F, G). The results showed that during liver aging, genes encoding inflammatory mediators and extracellular matrix components were progressively upregulated. This age-associated molecular dysregulation fosters a pro-inflammatory hepatic microenvironment and aberrant ECM remodeling, which mechanistically contribute to hepatic stellate cell activation and the initiation of de novo fibrogenesis, thereby accelerating the onset and progression of liver fibrosis^19^.

### Aging gene-derived scoring system maps functional heterogeneity in hepatic senescent cells

**Fig. 2:**
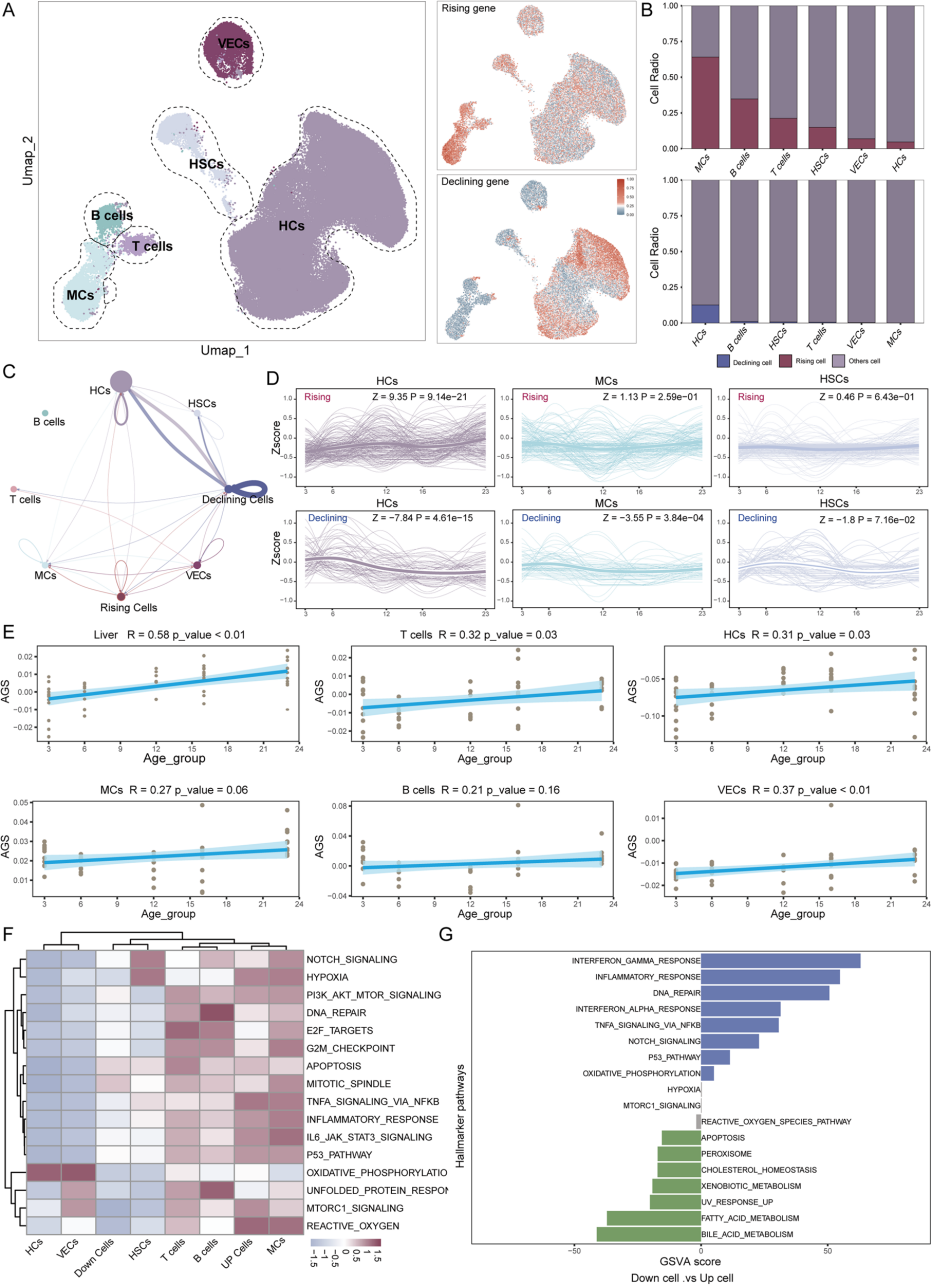
Single-cell landscape of aging-associated transcriptional dynamics and intercellular communication in the liver. **A** UMAP projection showing the annotation of major liver single-cell populations. Right panels: spatial distribution of AUCell scores for aging-related gene modules with increasing trends (top) and decreasing trends (bottom). **B** Proportional distribution of rising and declining cells within each major liver cell type. **C** Cell–cell communication network depicting inferred signaling interactions among declining cells, rising cells, and other cell populations, with edge thickness proportional to communication strength. **D** Age-dependent expression dynamics of aging trend genes in HCs, MCs, and HSCs, modeled using locally estimated scatterplot smoothing (LOESS) regression. **E** Correlation analysis between aging gene score (AGS) and chronological age across different liver cell types. **F** Heatmap showing functional enrichment profiles of rising and declining cells relative to other cell populations. **G** Bar plot summarizing differential enrichment of biological processes between rising and declining cells.

To evaluate the utility of senescence-associated genes in assessing liver aging at single-cell resolution, we analyzed a cross-sectional single-cell RNA-seq dataset of mouse liver spanning five age time points (GSE247719). Six major cell types were identified based on canonical marker gene expression: hepatocytes (HCs), hepatic stellate cells (HSCs), vascular endothelial cells (VECs), myeloid cells (MCs), T cells, and B cells (Fig. 2A, Fig. S1A). Using the senescence trend gene score, we found that upregulated senescence-associated genes were predominantly enriched in myeloid cells, whereas downregulated genes were primarily expressed in hepatocytes (Fig. 2A). This cell-type-specific pattern is consistent with recent single-cell studies reporting senescence-associated features in aged myeloid cells and their association with hepatic inflammation^20^.

We defined the top 10% of cells with the highest AUCell scores for upregulated and downregulated senescence-associated gene sets as “Rising cells” and “Declining cells,” respectively. Cells enriched for upregulated senescence-associated genes (Rising cells) were largely composed of myeloid cells, whereas those enriched for downregulated genes (Declining cells) were almost exclusively hepatocytes (Fig. 2B). To further explore whether cellular senescence is accompanied by alterations in intercellular communication, we performed analysis using CellChat. Overall, the inferred intercellular communication probability exhibited a declining trend with age, with relatively higher probabilities observed in young mice and lower probabilities in older mice. The magnitude of this decrease, however, varied across different cell-type pairs (Fig. 2C; Fig. S1B–D). Notably, the FN1 signaling pathway, which is closely associated with fibrotic processes, displayed the highest inferred communication probability in the oldest (23-month-old) samples, suggesting a potential age-dependent increase in fibrosis-related signaling (Fig. S1E). Consistent with these observations, temporal analysis showed that the expression of upregulated aging-related genes increased with age in HCs and HSCs, whereas only a modest, non-significant increase was observed in MCs. In contrast, downregulated genes exhibited a significant decline with age in HCs and MCs (Fig. 2D), indicating coordinated transcriptional remodeling of parenchymal and immune compartments during liver aging.

To quantify cellular aging dynamics at single-cell resolution, we defined the AGS as the difference between the AUCell scores of upregulated and downregulated aging trend genes. The AGS exhibited a significant positive correlation with chronological age at both the tissue and cell type levels (Fig. 2E), highlighting widespread transcriptional remodeling during liver aging. To further investigate the functional states of distinct cellular subpopulations, we compared Rising cells and Declining cells to other cell populations. Rising cells displayed strong transcriptional similarity to myeloid immune cells and were significantly enriched for pathways related to immune activation and inflammatory responses (Fig. 2F). These results are consistent with previous studies showing that aging-associated activation of hepatic myeloid cells contributes to liver fibrosis through pro-inflammatory signaling^21^. In contrast, Declining cells were functionally associated with cell cycle regulation and showed significant enrichment in pathways such as spindle assembly and mitosis, suggesting a proliferative phenotype characteristic of a potentially younger or regenerating cell state. Further differential pathway enrichment analysis revealed that Rising cells were strongly associated with immune-inflammatory signaling, including NF-ƙB and p53 pathways, whereas Declining cells were enriched for metabolic processes essential for hepatic parenchymal function, such as bile acid and fatty acid metabolism (Fig. 2G). These findings suggest that a reciprocal shift between immune activation and metabolic function may represent a core mechanism underlying liver aging and fibrosis progression.

### Senescence gene score reveals a link between liver aging and fibrosis

**Fig. 3:**
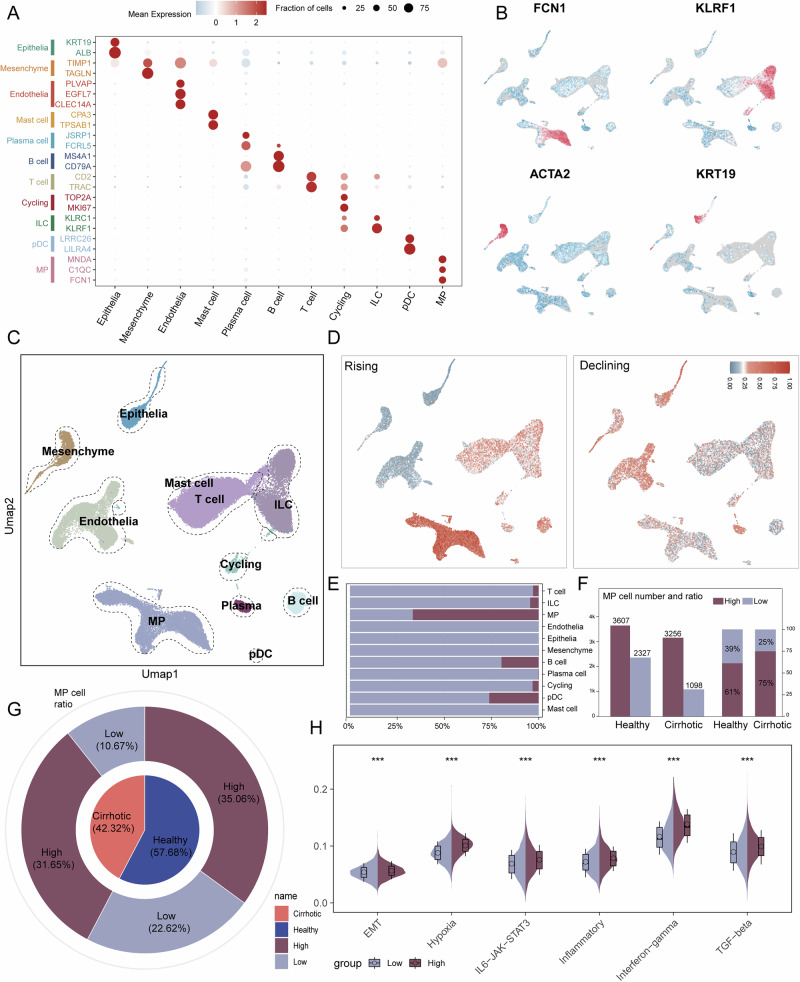
Single-cell characterization of aging-associated transcriptional states in non-parenchymal liver cells. **A** Dot plot showing the average expression of canonical marker genes across 11 major liver cell types. **B** UMAP feature plots displaying the expression patterns of representative marker genes across annotated cell populations. **C** UMAP projection illustrating the annotation of single-cell populations derived from fibrotic liver samples. **D** UMAP visualization of AUCell scores for genes with increasing and decreasing aging trends, with darker red indicating higher scores. **E** Bar plot showing the proportion of high-AGS and low-AGS cells across different cell types. **F** Bar plot summarizing the number and proportion of high- and low-AGS cells in healthy and cirrhotic liver samples. **G** Nested donut charts depicting the distribution of high-AGS and low-AGS cells within each major cell type. **H** Violin plots comparing functional enrichment scores between high-AGS and low-AGS cells.

To investigate aging-associated transcriptional dynamics in immune and stromal cells and to determine whether aging trend genes are associated with pathological liver fibrosis at single-cell resolution, we analyzed the non-parenchymal cell-enriched single-cell RNA-seq dataset GSE136103, which includes healthy and cirrhotic liver samples. Based on canonical marker gene expression, we annotated 11 major cell types, including epithelial cells, mesenchymal cells, endothelial cells, mast cells, B cells, plasma cells, T cells, innate lymphoid cells (ILCs), mononuclear phagocytes (MP), plasmacytoid dendritic cells (pDCs), and circulating cells (Fig. 3A–C). Subsequently, we calculated the AGS for each cell and stratified cells into high- and low-AGS groups, comparing their distributions across different cell types (Fig. 3D, E). Notably, MP represented the highest proportion of high-AGS cells, consistent with previous findings that hepatic macrophages are key mediators of aging-related inflammation and fibrosis via the secretion of TGF-β and other profibrotic cytokines^22^. Further analysis revealed that the proportion of high-AGS MP was significantly higher in cirrhotic livers than in healthy controls, reinforcing a close association between macrophage senescence and fibrosis progression (Fig. 3F, G).

These results highlight a potential mechanistic connection between liver aging and fibrosis, suggesting that MP-driven immunosenescence and fibroinflammatory remodeling are central contributors. Further functional enrichment analysis revealed that high-AGS cells were significantly enriched in pathways related to hypoxia, immune activation, and TGF-β signaling—canonical features of the fibrotic microenvironment (Fig. 3H). These pathways are known to drive hepatic stellate cell activation, extracellular matrix deposition, and chronic injury responses in the liver^23^, indicating that the AGS captures not only chronological aging but also the pathological hallmarks of fibrotic transformation.

### High AGS states accumulate along macrophage differentiation trajectories in cirrhotic liver

**Fig. 4:**
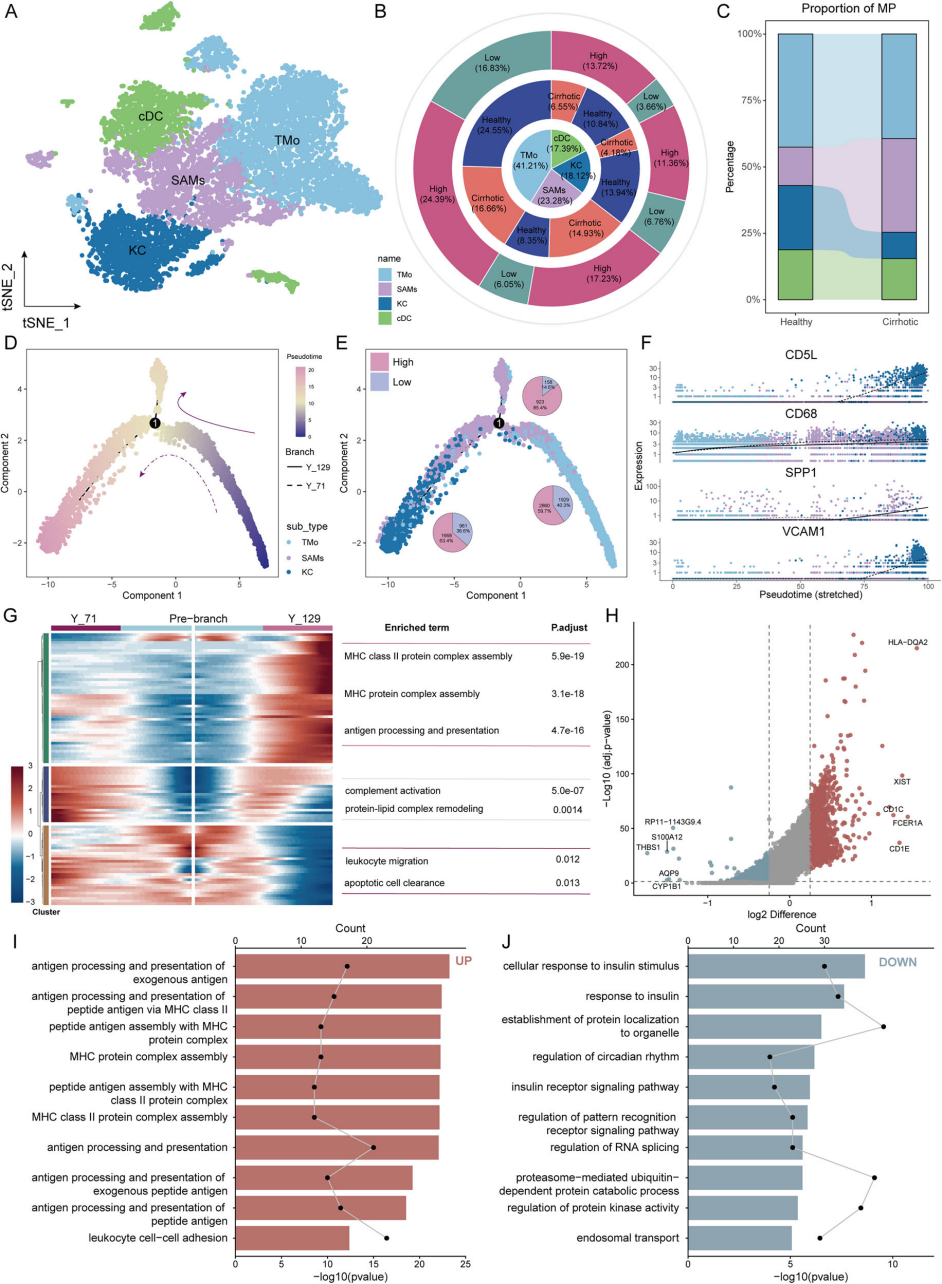
Heterogeneity and differentiation trajectories of macrophage subtypes during liver aging and fibrosis. **A** t-SNE plot showing the distribution of MP subtypes. **B** Nested donut charts illustrating the proportions of the four MP subtypes across high-AGS and fibrosis groups. **C** Bar plot showing the relative proportions of the four MP subtypes in healthy and cirrhotic liver samples. **D** Macrophage differentiation trajectory colored by pseudotime. **E** Differentiation trajectory colored by macrophage subtypes. **F** Expression dynamics of selected liver aging–related genes along distinct pseudotime branches. **G** Heatmap of DEGs along the pseudotime trajectory with associated Gene Ontology (GO) enrichment terms. **H** Volcano plot showing differential gene expression between high-AGS and low-AGS macrophage populations. GO enrichment analysis of upregulated (**I**) and downregulated (**J**) genes between high-AGS and low-AGS macrophage groups.

Mounting evidence suggests that mononuclear phagocytes play a pivotal role in liver aging and fibrosis. To further delineate regulatory differences among MP subtypes in fibrosis, we performed re-clustering analysis and, based on canonical marker genes, classified MP into four major subpopulations: tissue monocytes (TMo), scar-associated macrophages (SAMs), Kupffer cells (KC), and classical dendritic cells (cDC) (Fig. 4A, Fig. S1F). We then assessed the distribution of aging-associated gene signatures across these subpopulations. Compared with healthy livers, the proportions of KC and TMo decreased in cirrhotic samples, whereas SAMs showed a marked increase; the proportion of cDC remained largely unchanged (Fig. 4B, C; Fig. S2A). These observations align with previous reports indicating that SAMs, through the expression of pro-fibrotic genes, represent a key cellular component contributing to liver fibrosis progression^24^.

To further elucidate the lineage relationships among macrophage subtypes, we performed pseudotime analysis on KC, TMo, and SAMs (Fig. 4D, E). KC and SAMs were predominantly located at the terminal branches of the trajectory, suggesting terminal differentiation states, whereas TMo were enriched at the initial stage. Notably, along the TMo-to-SAM branch, high-AGS cells were more prevalent than in other branches, and their proportion increased progressively along the pseudotime trajectory, suggesting a dynamic association between senescence-associated transcriptional features and macrophage differentiation.

To further characterize the biological significance of AGS, we examined the dynamics of multiple gene signatures along the trajectory, including canonical senescence (SenMayo), DNA damage, cell cycle, inflammation, and fibrosis-related gene sets. While AGS showed significant associations with classical senescence signatures, it exhibited a stronger correlation with inflammatory and fibrotic programs. These findings suggest that AGS may represent a composite transcriptional feature integrating intrinsic senescence-related processes with microenvironment-induced inflammatory and fibrotic responses (Fig. S2B, C). Consistently, within the SAM population, cells with high AGS displayed significantly higher expression of inflammation- and fibrosis-related gene signatures compared to low-AGS cells. Notably, during differentiation from TMo to SAMs, expression of the pro-fibrotic gene SPP1 gradually increased (Fig. 4F), consistent with previous reports linking high *SPP1* expression to pro-fibrotic macrophage activity^25^. Functionally, the TMo-to-SAMs trajectory was enriched for inflammatory response pathways, whereas the TMo-to-KC trajectory was primarily associated with complement activation (Fig. 4G).

Comparison of gene expression between high- and low-AGS macrophages revealed that upregulated genes in the high-AGS group, including *FCER1A* and *CD1E*, are established markers of inflammation and pro-fibrotic activity (Fig. 4H). Enrichment analysis further showed that upregulated genes were predominantly involved in inflammation-related functions, such as MHC class II protein complex assembly and leukocyte adhesion, whereas downregulated genes were mainly associated with fundamental cellular processes, including RNA splicing and endocytosis (Fig. 4I, J). Collectively, these results provide multi-layered evidence that macrophage lineage-specific transcriptional programs contribute to liver aging and fibrosis progression.

### Characterization of aging-related molecular subtypes in fibrosis

**Fig. 5:**
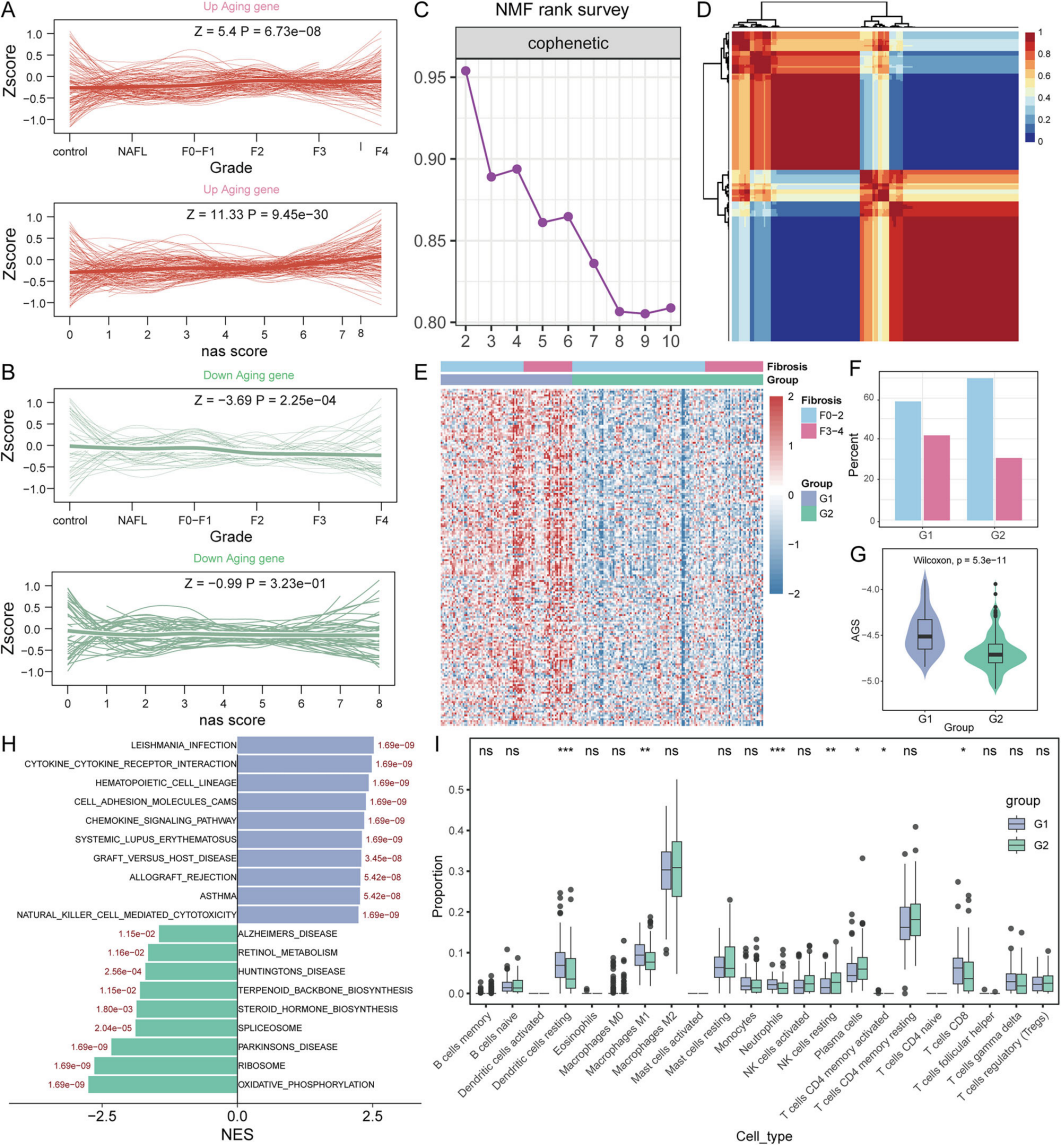
Aging-associated molecular subtypes and their relationship with liver fibrosis severity. **A** Expression trends of upregulated senescence-related genes across liver fibrosis stages and NAFLD activity score (NAS). **B** Expression trends of downregulated senescence-related genes across liver fibrosis stages and NAS. **C** Evaluation of non-negative matrix factorization (NMF) clustering stability using cophenetic correlation coefficients for k values ranging from 2 to 10; *k* = 2 was selected as the optimal number of clusters. **D** NMF consensus matrix heatmap for *k* = 2, showing robust sample stratification. **E** Heatmap illustrating the expression patterns of liver-specific aging genes across the two NMF-defined clusters and fibrosis stages. **F** Proportional distribution of samples across fibrosis stages within each NMF cluster. **G** Distribution of aging gene scores (AGS) between the two NMF clusters. **H** Bar plot summarizing Gene Set Enrichment Analysis (GSEA) results for the two NMF clusters. **I** Distribution and statistical comparison of 22 immune cell populations inferred by CIBERSORT between the two NMF clusters. Statistical significance is indicated as follows: ****p < 0.0001; ***p < 0.001; **p < 0.01; *p < 0.05; ns not significant (*p* > 0.05).

To validate the role of liver aging-related genes in the progression of liver fibrosis, we performed a multidimensional analysis using a human nonalcoholic fatty liver disease (NAFLD) liver transcriptomic dataset (GSE135251). This dataset includes patient samples spanning different fibrosis stages and NAFLD Activity Scores (NAS), a histological scoring system that assesses disease severity based on steatosis, inflammation, and hepatocellular injury. Locally weighted scatterplot smoothing (LOESS) regression analysis revealed that the expression of upregulated aging-associated genes significantly increased with fibrosis stage and NAS. In contrast, downregulated genes exhibited a decreasing trend; however, this trend did not reach statistical significance with respect to NAS (Fig. 5A, B). These opposing expression patterns indicate that aging-associated transcriptional programs are closely associated with the pathological progression of liver fibrosis.

To further dissect the molecular heterogeneity underlying these patterns, we employed non-negative matrix factorization (NMF) based on the identified aging trend genes to stratify patients into two distinct molecular subtypes, G1 and G2 (Fig. 5C, D). Clinical association analysis revealed that the G1 subtype contained a significantly higher proportion of patients with advanced fibrosis (F3–F4) compared with the G2 subtype, whereas G2 was enriched for patients with early-stage fibrosis (F0–F2) (Fig. 5E, F; Fig. S2D). Consistent with this pattern, the G1 subtype exhibited higher AGS values for aging-associated signatures than G2 on average, despite notable intra-group heterogeneity (Fig. 5G). These results support an association between transcriptional aging programs and fibrosis severity.

Gene Set Enrichment Analysis revealed that the G1 subtype was characterized by robust activation of immune and inflammatory pathways, including Toll-like receptor signaling and NF-κB signaling, which are known to mediate hepatic inflammation and fibrosis by promoting macrophage activation and pro-inflammatory cytokine release. In contrast, the G2 subtype showed enrichment in metabolic and proteostatic pathways, such as fatty acid metabolism, oxidative phosphorylation, and proteasome function, reflecting a more quiescent and hepatocyte-centered transcriptomic profile (Fig. 5H). Immune infiltration analysis using CIBERSORT further highlighted the inflammatory characteristics of the G1 subtype, with a significant enrichment of pro-inflammatory M1 macrophages. In contrast, M2 macrophages and resting NK cells were significantly enriched in the G2 subtype, while resting dendritic cells were relatively increased in G1 (Fig. 5I). These patterns are consistent with the established association of M1-polarized macrophages with liver inflammation and fibrosis^26^.

### Identification of fibrosis biomarkers based on aging-related genes

**Fig. 6:**
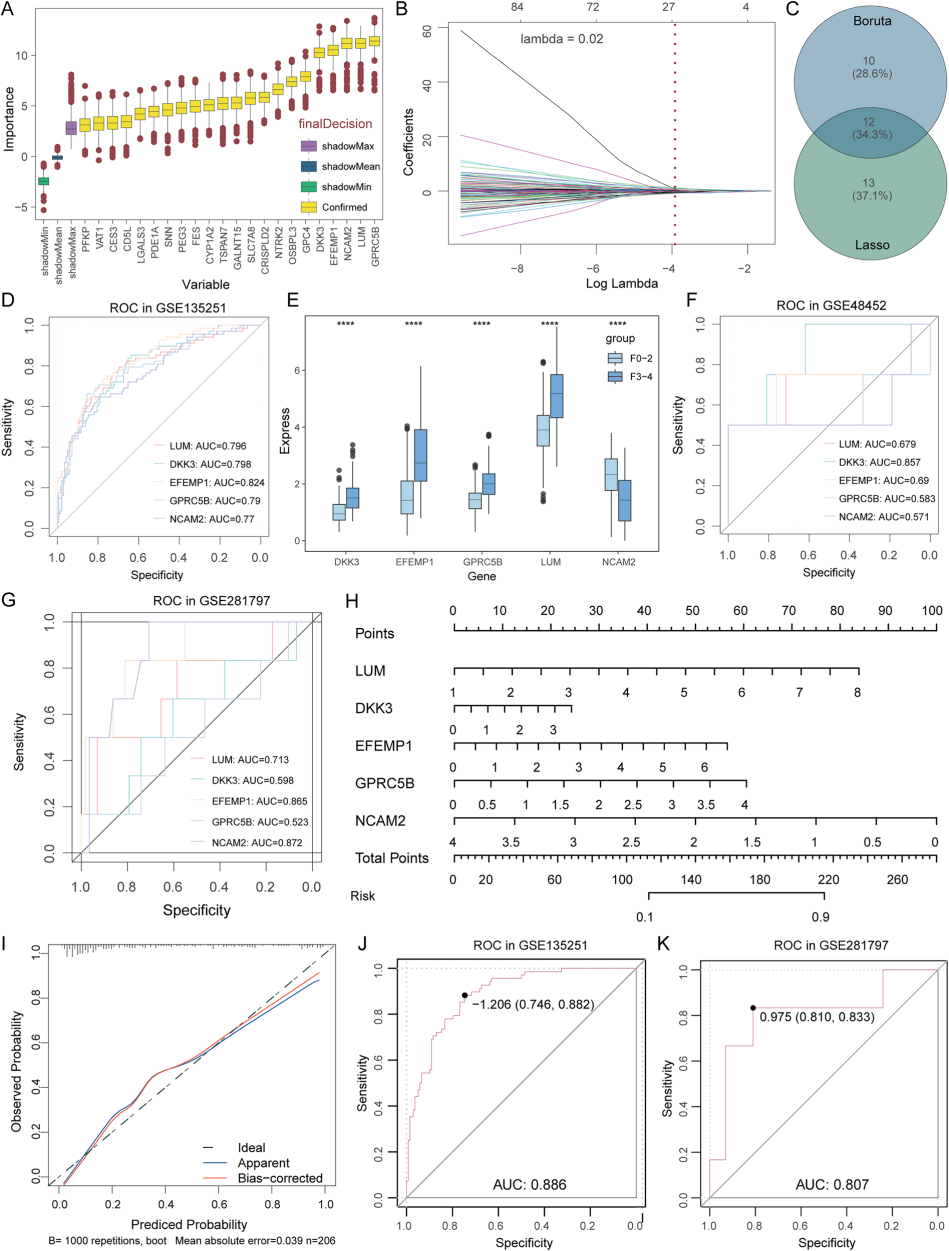
Identification and validation of fibrosis-associated biomarkers and construction of a predictive nomogram. **A** Feature selection results obtained using the Boruta algorithm. **B** Regression coefficient trajectories of genes selected by the Lasso algorithm. **C** Venn diagram showing the overlap between genes selected by Boruta and Lasso methods. **D** ROC curves evaluating the predictive performance of candidate biomarkers in the training dataset. **E** Expression patterns of the selected biomarkers across different fibrosis stages. **F**, **G** ROC curves assessing the performance of these biomarkers in the independent validation datasets GSE48452 and GSE281797. **H** Nomogram integrating the selected biomarkers for predicting fibrosis stage. **I** Calibration curves comparing predicted versus observed fibrosis stages to evaluate model accuracy. **J**, **K** ROC curves demonstrating the predictive performance of the nomogram in the training (GSE135251) and validation (GSE281797) datasets.

To identify robust biomarkers, we conducted feature selection using two different machine learning methods based on liver specific aging-related genes in the training dataset GSE135251. First, we applied the Boruta algorithm for feature selection, which calculated the importance of each gene feature and identified 22 key features (Fig. 6A). Additionally, we constructed a Lasso regression model, retaining 25 significant gene features (Fig. 6B; Fig. S2E). Among the features identified by both algorithms, we found 12 overlapping genes: *LUM*, *PEG3*, *TSPAN7*, *DKK3*, *EFEMP1*, *GALNT15*, *GPRC5B*, *NCAM2*, *SNN*, *FES*, *SLC7A8*, and *VAT1* (Fig. 6C). Of these, five genes (*LUM*, *DKK3*, *EFEMP1*, *GPRC5B*, and *NCAM2*) demonstrated an area under the ROC curve (AUC) greater than 0.77 and were selected as biomarkers for fibrosis (Fig. 6D). These five biomarkers also exhibited significant expression differences between early and late fibrosis samples (Fig. 6E). Inter-dataset heterogeneity was evident, as the top-performing biomarker differed across cohorts. In GSE48452, *DKK3* achieved the highest discriminative performance (AUC = 0.857), whereas in GSE281797, *NCAM2* (AUC = 0.872) and *EFEMP1* (AUC = 0.865) demonstrated superior predictive accuracy (Fig. 6F, G). This variability highlights the inherent limitations of single-marker–based models and underscores the necessity of integrating multiple biomarkers to achieve robust and generalizable predictive performance across independent cohorts.

To comprehensively assess the predictive performance of the identified biomarkers for advanced fibrosis, we constructed a nomogram model using the training cohort (GSE135251) (Fig. 6H). Each biomarker was assigned a weighted score, and the cumulative score was used to estimate the individual probability of advanced fibrosis. Receiver operating characteristic (ROC) curve analysis demonstrated excellent discriminative ability of the model in the training set (Fig. 6I), while calibration analysis indicated a high concordance between the predicted risks and the observed outcomes (Fig. 6J). Importantly, the model retained robust predictive accuracy in an independent validation cohort (GSE281797) (Fig. 6K). Collectively, these results indicate that the integrated nomogram outperforms individual biomarkers in stratifying the clinical risk of advanced fibrosis.

### Drug discovery and molecular docking of food-medicine homologous compounds in liver aging and fibrosis

**Fig. 7:**
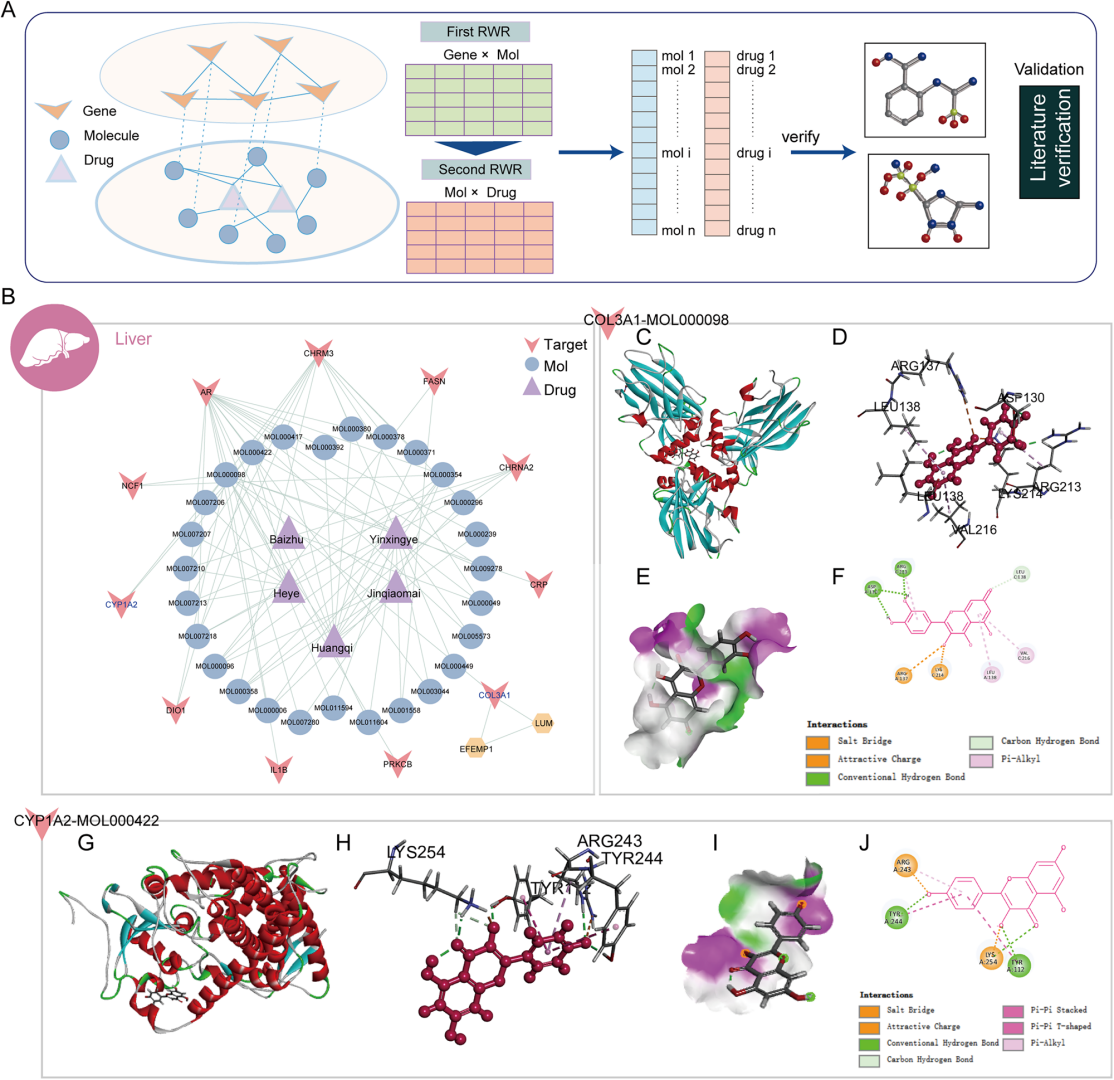
Drug screening and molecular docking analysis of liver aging-associated candidate compounds. **A** Schematic illustration of the construction workflow for the liver aging-associated drug screening model. **B** Drug ranking results based on liver aging trend genes. The top five candidate drugs, along with their corresponding small molecules and target genes, were identified and visualized through network analysis. **C** Predicted binding conformation of MOL000098 within the *COL3A1* protein. **D** Binding pocket structure showing hydrogen bond donors and acceptors. **E** Three-dimensional interaction diagram depicting the binding between MOL000098 and *COL3A1* amino acid residues. **F** Two-dimensional interaction map illustrating the interactions between MOL000098 and residues of *COL3A1*. **G** Predicted binding conformation of MOL000422 within the *CYP1A2* protein. **H** Binding pocket structure showing hydrogen bond donors and acceptors. **I** Three-dimensional interaction diagram depicting the binding between MOL000422 and *CYP1A2* amino acid residues. **J** Two-dimensional interaction map illustrating the interactions between MOL000422 and residues of *CYP1A2*.

Aging is a complex, multifactorial process characterized by the progressive decline of cellular, tissue, and systemic functions. In the context of anti-aging interventions, Chinese medicinal herbs with both medicinal and dietary applications—classified as food-medicine homologous substances—have demonstrated great potential due to their edible safety profile, broad bioactivity, and multi-target, systems-level regulatory effects^27,28^. In our study, we found that liver aging trend genes exhibit pronounced time-dependent expression patterns, suggesting that therapeutic strategies targeting these genes may offer sustained benefits in delaying the aging process. To systematically identify food-medicine homologous Chinese herbs with potential anti-aging effects on the liver, we developed a screening framework based on organ-specific aging trend genes and applied a restarted random walk (RWR) algorithm to construct a drug-target association network (Fig. 7A). Using liver aging-related genes as input and integrating information from curated herb-target databases, we built a multilayered interaction network comprising food-medicine homologous herbs, small molecules, and their respective targets. Anti-aging scores for each compound were calculated based on the adjacency matrix and a two-step random walk algorithm (Table 1).

**Table 1:** Drug scores calculated by the random walk with restart (RWR) algorithm

Drugs	Score
Baizhu	0.0138475172403155
Jinqiaomai	0.00621490806616577
Yinxingye	0.00486269095577692
Heye	0.00477589677054505
Huangqi	0.00470499716265722

The score represents the steady-state probability of each drug in the RWR network, indicating its relative association with aging-related genes. Drugs were ranked by RWR score, and the top five candidates are shown.

The results identified several high-ranking herbs with both medicinal and edible properties, including *Astragalus membranaceus* (Huangqi), *Fagopyrum dibotrys* (Jinqiaomai), *Atractylodes macrocephala* (Baizhu), *Ginkgo biloba* (Yinxingye), and *Nelumbo nucifera* (Heye) (Fig. 7B). *Astragalus membranaceus*, a traditional Qi-tonifying herb, has been shown to enhance telomerase activity and improve immune function through its active component TA-65, thereby delaying immune system aging^29,30^. *Fagopyrum dibotrys* is rich in flavonoids such as rutin and exhibits antioxidant, lipid-lowering, glucose-regulating, and microcirculation-enhancing effects, which may contribute to mitigating aging-related liver fibrosis via metabolic modulation^31^. *Atractylodes macrocephala* and *Nelumbo nucifera* are widely used in traditional diets and have been included in the official Chinese catalog of food-medicine homologous substances. Their roles in delaying liver functional decline and improving lipid metabolism warrant further investigation^32,33^.

To evaluate the interactions between the five candidate drugs and their putative target genes, we first conducted molecular docking analyses using Discovery Studio. Binding affinity was assessed using the −CDOCKER ENERGY score, with higher values indicating more stable ligand–protein interactions. Among all tested combinations, compound MOL000098 exhibited the highest binding affinity toward the target protein COL3A1 ( − CDOCKER ENERGY = 60.38), suggesting a particularly stable interaction (Fig. 7C–J; Table 2). These results support the potential relevance of *Fagopyrum dibotrys*, *Ginkgo biloba*, *Nelumbo nucifera*, and *Astragalus membranaceus* in modulating molecular processes related to liver aging and fibrosis.

**Table 2:** Molecular docking information of components and targets

Targets	PDB	Components	-CDOCKER ENERGY	Drugs
COL3A1	4AE2	MOL000098	60.3799	Jinqiaomai, Yinxingye, Heye, Huangqi
CYP1A2	2HI4	MOL000422	47.8811	Yinxingye, Heye, Huangqi
IL1B	1L2H	MOL000098	45.8752	Jinqiaomai, Yinxingye, Heye, Huangqi
CRP	1B09	MOL000098	45.6266	Jinqiaomai, Yinxingye, Heye, Huangqi
PRKCB	2I0E	MOL000098	38.6846	Jinqiaomai, Yinxingye, Heye, Huangqi
AR	1XOW	MOL000422	37.4525	Yinxingye, Heye, Huangqi
NCF1	1KQ6	MOL000354	37.4261	Jinqiaomai, Yinxingye, Heye, Huangqi
FASN	3TJM	MOL000096	30.8728	Jinqiaomai, Yinxingye, Heye
CHRNA2	5FJV	MOL007210	10.7373	Heye
CHRM3	8EA0	MOL007207	3.52674	Heye

-CDOCKER ENERGY indicates predicted binding affinity, with higher values representing stronger interactions. PDB IDs refer to protein structures used for docking.

To further explore indirect associations between the five fibrosis-related biomarkers identified in this study and anti-aging drugs, we performed a random walk with restart (RWR) analysis on the drug–gene interaction network. Although these biomarkers are not established direct drug targets, their network proximity to a canonical drug target (*COL3A1*) prioritized *Fagopyrum dibotrys and Ginkgo biloba* as the most strongly associated candidates (Supplementary Data 1). We then performed molecular docking analyses between the five biomarkers and three active small molecules derived from *Fagopyrum dibotrys*. All ligand–protein pairs showed favorable binding potentials, with docking scores ranging from 14 to 68 (Fig. S3; Supplementary Data 2), suggesting that these biomarkers may represent plausible interaction partners of the tested compounds. Finally, molecular dynamics simulations indicated that ligand binding could induce localized conformational stabilization of target proteins. Such as, MOL00354 appeared to promote the formation of a relatively stable rigid structural region spanning residues 217-236 of the *NCAM2* protein, providing structural insights into its potential mode of action (Supplementary Data 3).

### Protective effects of Jinqiaomai-containing serum on hepatocytes and macrophages

**Fig. 8:**
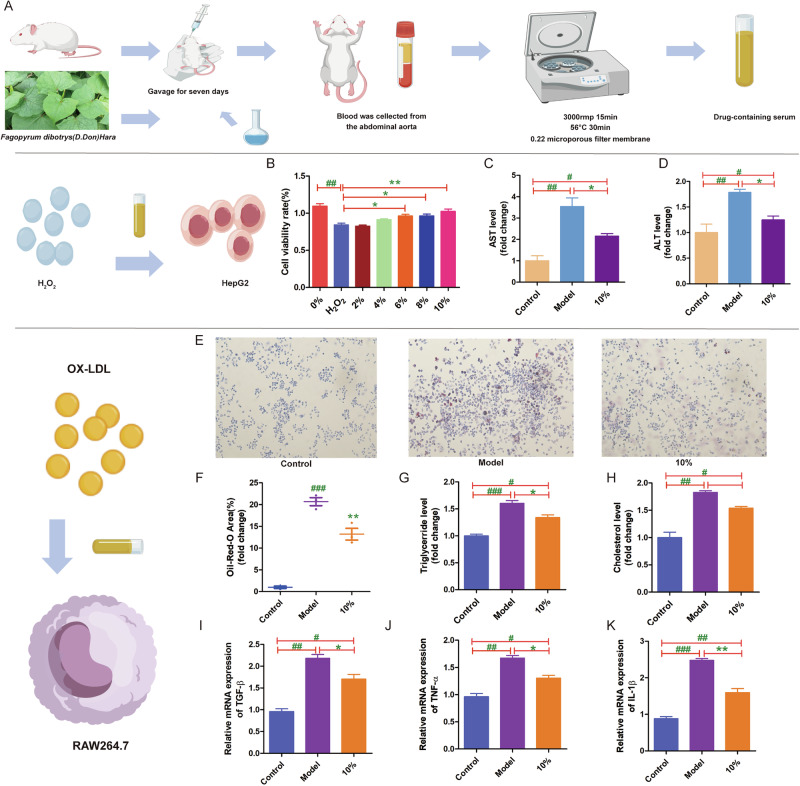
Effects of *Fagopyrum dibotrys* (JQM) extract on liver function and macrophage foam cell formation. **A** Schematic illustration of the preparation process of JQM extract. **B** Effects of different concentrations of JQM-containing serum on HepG2 cell viability, measured by CCK-8 assay. Effects of JQM-containing serum on liver injury biomarkers: **C** aspartate aminotransferase (AST) and **D** alanine aminotransferase (ALT). **E** Representative images of Oil Red O staining in macrophages from the normal group, control group, and 10% JQM-treated group, showing reduced foam cell formation after JQM treatment. **F** Quantification of Oil Red O staining intensity in macrophages, indicating a significant decrease in lipid accumulation in the drug-treated group. Bar charts showing the intracellular cholesterol (**G**) and triglyceride (**H**) content in different groups. Treatment with JQM-containing serum significantly reduced lipid accumulation compared with the model group. Expression levels of fibrosis-related factor TGF-β (**I**) and pro-inflammatory cytokines TNF-α (**J**) and IL-1β (**K**) across groups, demonstrating attenuation of inflammatory and pro-fibrotic responses in the 10% JQM-treated group. Data are presented as mean ± SD. #*p* < 0.05, ##*p* < 0.005, ###*p* < 0.001 compared with the control group; *p < 0.05, **p < 0.005 compared with the 10% JQM-treated group.

To investigate the cytoprotective effects of serum containing *Fagopyrum dibotrys* (JQM), we first evaluated its activity in a HepG2 cell model. Cells were pretreated with increasing concentrations (2–10%) of JQM-containing rat serum for 24 h, followed by exposure to oxidative stress induced by 150 μM ${H}_{2}{O}_{2}$ for an additional 24 h (Fig. 8A). CCK-8 assays showed that pretreatment with 6%, 8%, and 10% JQM serum significantly improved cell viability compared with the ${{\rm{H}}}_{2}{{\rm{O}}}_{2}$-treated model group, with the 10% concentration conferring the strongest protective effect (Fig. 8B). Accordingly, 10% JQM serum was used in subsequent experiments.

We next assessed intracellular alanine aminotransferase (ALT) and aspartate aminotransferase (AST) activities as indicators of hepatocellular injury. ${{\rm{H}}}_{2}{{\rm{O}}}_{2}$ exposure markedly increased ALT and AST activities, whereas pretreatment with 10% JQM serum significantly attenuated these elevations (Fig. 8C, D), indicating effective protection against oxidative stress–induced hepatocyte damage.

To assess the effect of JQM-containing serum on lipid accumulation in macrophages, we measured intracellular total cholesterol (TC) and triglyceride (TG) levels and performed Oil Red O staining. Compared with the ox-LDL stimulation group, treatment with JQM-containing serum markedly reduced intracellular lipid accumulation (Fig. 8E–H). Ox-LDL induction (50 μg/mL) significantly increased TC and TG levels, whereas pretreatment with 10% JQM-containing serum effectively reversed this effect, significantly lowering both lipid measures. In addition, we assessed the expression of pro-inflammatory cytokines (IL-1β and TNF-α) and fibrosis-related factors (TGF-β). These markers were significantly upregulated following ox-LDL stimulation and were markedly reduced upon JQM treatment, indicating attenuation of inflammatory and pro-fibrotic activation. Considering that macrophage foaming is a critical driver of chronic inflammation and fibrosis^34^, these findings indicate that *Fagopyrum dibotrys* may exert anti-inflammatory and anti-fibrotic effects by modulating macrophage phenotype, further highlighting its potential therapeutic value as a food-medicine homology substance.

## Discussion

As a central metabolic organ, the liver plays a pivotal role in maintaining systemic homeostasis. With aging, hepatic function progressively declines, increasing susceptibility to age-related diseases such as MASLD, cirrhosis, and hepatocellular carcinoma^2^. These pathological changes are often driven by chronic inflammation, immune dysregulation, and extracellular matrix remodeling^35^. In this study, we constructed a comprehensive framework that integrates time-series transcriptomic analysis, scRNA-seq, machine learning, and experimental validation to delineate the molecular landscape of liver aging and its mechanistic link to fibrosis.

Our study comprehensively characterized liver aging dynamics at both transcriptomic and single-cell levels. The identification of 252 aging trend genes revealed a core regulatory network involving immune activation, extracellular matrix remodeling, and metabolic repression—hallmarks of hepatic aging. The upregulation of genes involved in immune-related signaling, such as MHC complex components and cytokines, suggests an age-dependent shift toward chronic, low-grade inflammation, which compromises hepatic immune tolerance and predisposes the liver to fibrotic transformation^36^

At single-cell resolution, we mapped the cellular heterogeneity of aging using an aging gene score. This analysis revealed that myeloid cells, particularly macrophages, were the primary reservoir of high AGS cells in aged and cirrhotic livers, aligning with their known role in secreting pro-fibrotic cytokines like TGF-β^11,37^. Re-clustering of mononuclear phagocytes (MPs) uncovered distinct subtypes with varying associations to fibrosis: scar-associated macrophages (SAMs) were markedly expanded in cirrhosis, whereas Kupffer cells (KCs) and tissue monocytes (TMo) were diminished. Pseudotime trajectory analysis further illuminated the evolutionary relationships among these subtypes. We observed that TMo cells, enriched at the trajectory origin, gave rise to terminal-state KCs and SAMs. The differentiation trajectory from TMo to SAMs was accompanied by a gradual increase in both the AGS and the expression of the pro-fibrotic gene *SPP1*, and was functionally enriched for inflammatory pathways. This compellingly links macrophage functional specialization (particularly toward a pro-fibrotic SAMs phenotype) with the accumulation of senescence-associated transcriptional states^38^.

Based on the aging-related gene set, we applied machine learning approaches to identify a biomarker panel comprising five genes—*LUM*, *DKK3*, *EFEMP1*, *GPRC5B*, and *NCAM2*—that exhibited strong diagnostic potential for distinguishing advanced liver fibrosis. The involvement of these genes in fibrotic processes is well supported by previous studies. For instance, *DKK3* is upregulated in injured and chronically failing livers, and its genetic deletion markedly alleviates liver injury and fibrosis in murine models^39^. *EFEMP1*, an extracellular matrix–associated protein, directly participates in ECM synthesis, degradation, and tissue remodeling, while *LUM* is a well-established fibrosis biomarker whose expression positively correlates with fibrosis severity^40,41^. Notably, our protein–protein interaction network analysis revealed that *LUM* and *EFEMP1* directly interact with the key drug target *COL3A1*, suggesting the existence of a coordinated regulatory network that may drive age-related fibrotic remodeling. Finally, we integrated these five biomarkers into a nomogram-based prediction model, which demonstrated robust and reproducible predictive performance across both training and independent validation cohorts. This integrated model outperformed individual biomarkers, providing a practical and reliable tool for clinical risk stratification of advanced liver fibrosis.

To explore potential therapeutic strategies, we implemented a drug discovery pipeline incorporating aging gene signatures and network-based screening. Focusing on food-medicine homologous herbs—natural products that serve both nutritional and medicinal roles—we identified several promising candidates, including *Fagopyrum dibotrys*, *Astragalus membranaceus*, and *Nelumbo nucifera*. These herbs are rich in bioactive compounds such as flavonoids, saponins, and polysaccharides, which exhibit anti-inflammatory, antioxidant, and immunomodulatory activities^28,32,42^. Using molecular docking, we confirmed high-affinity binding between active compounds and fibrosis-related targets such as *COL3A1*, supporting their potential efficacy in modulating liver aging and fibrotic signaling pathways. Importantly, in vitro validation showed that serum containing Jinqiaomai extract conferred significant cytoprotection against oxidative stress in HepG2 cells and effectively suppressed pro-fibrotic macrophage foaming in RAW264.7 cells, providing functional support for its therapeutic potential.

Despite these promising findings, our study has several limitations. First, many analyses were based on publicly available datasets, which may introduce potential bias. Second, although we performed in vitro validation using an ox-LDL–induced foam macrophage model, this system does not fully recapitulate fibrosis-associated cellular interactions, particularly macrophage–hepatic stellate cell (HSC) crosstalk. While our results suggest that foam macrophage formation is accompanied by inflammatory and pro-fibrotic activation and that AGS-guided compounds exert protective effects in this context, more physiologically relevant models (e.g., co-culture systems) are needed for further validation. Finally, additional in vivo experiments and clinical data are required to substantiate the therapeutic potential of these findings.

In conclusion, our study provides a multi-scale perspective on liver aging, integrating molecular signatures, cellular dynamics, biomarker prediction, and natural product-based therapeutic screening. By establishing a link between immune senescence and fibrotic remodeling, and identifying both predictive biomarkers and food-medicine homologous interventions, we offer a theoretical and experimental foundation for the early diagnosis and personalized treatment of aging-associated liver diseases.

## Methods

### Data collection and preprocessing

In this study, multiple transcriptomic datasets related to liver aging and fibrosis were downloaded from the Gene Expression Omnibus (GEO) database^43^. Bulk RNA sequencing datasets included GSE132040^44^, which comprises 55 mouse liver tissue samples spanning 10 time points from 1 to 27 months of age and was used to identify liver-specific aging-related genes. GSE135251^45^, containing liver tissue samples from 206 patients with metabolic dysfunction-associated fatty liver disease (MASLD) across fibrosis stages F0 to F4, was used to evaluate the association between aging-related genes and fibrosis severity. In addition, GSE48452^46^ and GSE281797, which include 25 and 94 liver tissue samples at different fibrosis stages, respectively, were used for independent validation of candidate biomarkers and diagnostic models.

In addition, we retrieved two single-cell RNA sequencing datasets, GSE247719^47^ and GSE136103^48^, from the Gene Expression Omnibus (GEO) database. The GSE247719 dataset is part of the PanSci mouse transcriptomic atlas and comprises a cross-sectional single-nucleus RNA sequencing dataset across different ages. The data were generated using EasySci, a combinatorial indexing–based single-nucleus RNA sequencing method optimized for large-scale profiling. The dataset includes mouse liver samples collected at 3, 6, 12, 16, and 23 months of age. The original dataset provides pre-annotated single-cell data with tissue-level annotations. Liver-derived cells were extracted based on these annotations, and subsequent analyses were performed on this subset. Standard preprocessing steps, including normalization and cell-type annotation, were applied to identify hepatocytes for downstream analyses. This dataset was used to investigate the impact of aging trend genes at the single-nucleus level and to validate the identification of senescence-associated cellular populations during liver aging. The GSE136103 dataset consists of single-cell RNA sequencing profiles generated using the 10x Genomics Chromium Single Cell 3’v2 platform, a droplet based single ell RNA sequencing method. It comprises samples from five cirrhotic and five normal livers and is enriched for non-parenchymal cells, enabling the investigation of aging-related transcriptional programs in the context of liver fibrosis at single-cell resolution. An overview of the dataset selection and analytical workflow is provided in Fig. S4.

### Bulk RNA-seq data preprocessing

All raw count matrices from bulk RNA-seq datasets underwent a two-step quality control and normalization procedure. First, genes with low expression levels—defined as those detected in fewer than 20% of samples—were excluded from downstream analyses. Subsequently, data normalization was performed using the edgeR^49^ package by applying counts per million (CPM) normalization followed by log2 transformation (log2-CPM). This normalization strategy corrects for differences in library size and sequencing depth, thereby enabling accurate and reliable comparisons of gene expression levels across samples.

### Identification of liver-specific aging genes

To identify liver-specific aging genes during the aging process, we performed time-series differential expression analysis using the R package ImpulseDE2^50^ based on the GSE132040 dataset. The function runImpulseDE2 was applied to analyze gene read counts across 10 aging time points in liver samples. Genes with a *p* < 0.05 were considered differentially expressed genes (DEGs).

Next, clustering analysis was conducted using the Mfuzz algorithm from the R package ClusterGVis (https://github.com/junjunlab/ClusterGVis)^51^. Based on the average expression levels of DEGs across 10 time points, the genes were grouped into nine clusters exhibiting distinct but internally coherent temporal expression patterns. Clusters showing highly similar stage-specific dynamics were further merged. To ensure the robustness of cluster assignments, we applied a membership threshold >0.8 to identify key genes within each cluster. Subsequently, we employed the Mann-Kendall non-parametric trend test^52^ to systematically evaluate monotonic temporal trends in gene expression. Using a significance cutoff of *p* < 0.05, genes showing consistent unidirectional changes (continuously increasing or decreasing) across multiple time points were identified as aging trend genes.

### Functional enrichment analysis

To investigate the molecular functions and key biological pathways associated with liver-specific aging-related genes, functional enrichment analyses were performed using the R package clusterProfiler^53^. Gene Ontology (GO) enrichment analysis was conducted across three categories, including biological processes (BP), molecular functions (MF), and cellular components (CC), to comprehensively characterize the functional attributes of aging-related genes. In parallel, Kyoto Encyclopedia of Genes and Genomes (KEGG) pathway enrichment analysis was performed to identify key signaling pathways potentially involved in liver aging and fibrosis. For both GO and KEGG analyses, a *p* < 0.05 was applied as the threshold for statistical significance. Enrichment results were visualized using the R package ggplot2, including bar plots and dot plots to display the most significantly enriched GO terms and KEGG pathways.

### Preprocessing of single-cell RNA sequencing data

To construct a single-cell atlas of liver aging and to delineate aging-associated transcriptional programs across cell types, we analyzed the GSE247719 dataset. In parallel, to investigate the relationship between aging and liver fibrosis at single-cell resolution, we analyzed the GSE136103 dataset. These two datasets were generated using distinct sequencing platforms: GSE247719 was produced using EasySci-based combinatorial indexing single-nucleus RNA sequencing (snRNA-seq), whereas GSE136103 was generated using the 10x Genomics Chromium droplet-based single-cell RNA sequencing (scRNA-seq) platform. Given the inherent platform-specific differences in library preparation and transcript capture, the two datasets were analyzed independently, and no direct cross-dataset integration was performed to minimize technical bias.

For GSE247719, which comprises snRNA-seq profiles from mouse livers at 3, 6, 12, 16, and 23 months of age, data preprocessing was conducted using the Seurat^54^ R package. Low-quality nuclei were excluded based on the following criteria: unique molecular identifier (UMI) counts <500, mitochondrial gene content >10%, or detection of fewer than 500 or more than 5000 genes. Genes expressed in fewer than three nuclei were removed. Raw UMI counts were log-normalized, and the top 2000 highly variable genes were identified using the FindVariableFeatures function. Principal component analysis (PCA) was performed for dimensionality reduction, followed by batch effect correction across samples using Harmony. Cell clustering and visualization were conducted using t-distributed stochastic neighbor embedding (t-SNE). Cell identities were assigned based on established canonical marker genes and reference annotations from the CellMarker database. For GSE136103, which includes scRNA-seq data from five healthy and five cirrhotic human livers generated using the 10x Genomics platform, the same quality control thresholds and preprocessing workflow were applied to ensure analytical consistency across datasets.

### Quantification of expression of aging trend genes

We assessed the activity of liver-specific senescence genes at the single-cell level using the R package AUCell. Positive AGS (Aging Gene Score) was derived from expression of gene clusters 6 and 8, which represent upregulated aging trends, while negative AGS was based on cluster 2, representing downregulated aging trends. Cells within the top 10% of AUCell scores for rising trend genes were defined as “Rising cells,” whereas those within the top 10% for declining trend genes were labeled as “Declining cells.” For each cell, the AGS was calculated as the difference between its positive and negative AGS values. Cells with AGS values in the top 15% were categorized into the high AGS group, and the remaining cells were assigned to the low AGS group.

### LOESS-based trajectory analysis of aging trend genes at the single-cell level

To evaluate whether the identified aging-associated genes display coherent age-dependent transcriptional dynamics at single-cell resolution, we performed trajectory analysis using the GSE247719 dataset. To mitigate the inherent sparsity and noise of single-cell RNA-seq data, gene expression profiles were aggregated at the donor and age-group levels to generate a pseudobulk expression matrix, thereby improving statistical robustness. Pseudobulk counts were normalized using the DESeq2 framework, including library size correction and variance-stabilizing transformation. Subsequently, Z-score standardization was applied to facilitate cross-sample comparability and reduce residual batch effects. For both upregulated and downregulated aging gene sets, locally estimated scatterplot smoothing (LOESS) regression^55^ was employed to model temporal expression trajectories, with chronological age treated as the independent variable and standardized median gene expression as the response. This strategy enabled the characterization and visualization of smooth, continuous age-related transcriptional trends of aging-associated genes across the liver single-cell landscape.

### CellChat-based cell–cell communication analysis

To systematically characterize dynamic changes in intercellular communication during liver aging, CellChat was employed to infer cell–cell communication networks from single-cell RNA sequencing (scRNA-seq) data. CellChat utilizes a curated ligand–receptor interaction database to estimate communication probabilities and to quantify the activity of signaling pathways. Briefly, CellChat objects were constructed using normalized gene expression matrices along with corresponding cell type annotations, stratified by age groups. Potential ligand–receptor interactions were then identified based on the CellChat database, and communication probabilities between cell types were computed using the built-in probabilistic framework. Significant intercellular communication events were subsequently identified, and pathway-level communication activities were inferred, enabling the reconstruction and comparison of cell–cell communication networks across different aging stages.

### Pseudotime trajectory analysis

We conducted pseudotime trajectory analysis of key cells using the R package Monocle^56^. The analysis incorporated marker genes from cell subpopulations identified through Seurat clustering, along with the raw expression counts of filtered cells. Individual cells within each population were projected onto a trajectory with a root and multiple branches. Dimensionality reduction and clustering were performed using the DDRTree algorithm, and cell differentiation states were determined using the orderCells function to construct a single-cell trajectory map.

### Non-negative matrix factorization (NMF) clustering of aging trend genes

To identify aging subtypes associated with liver aging, we performed unsupervised NMF clustering using liver-specific aging trend genes^57^. The analysis was performed on the GSE135251 dataset with normalized gene expression profiles as input. NMF decomposition was carried out using the NMF package in R, and a range of rank parameters (k) was evaluated to determine the optimal number of subtypes. Clustering stability was assessed by examining the cophenetic correlation coefficient across different k values. The optimal cluster number was defined at the point where the cophenetic coefficient exhibited a marked decline, indicating reduced clustering stability. Based on this criterion, *k* = 2 was selected as the most robust solution, and samples were accordingly assigned to Group 1 (G1) and Group 2 (G2). To further validate the robustness of this classification, we inspected the consensus heatmap, which consistently supported the presence of two distinct age-related molecular subtypes.

### Clustering characteristic analysis based on aging genes

Using liver-specific aging trend genes, we applied the ssGSEA algorithm to calculate the aging gene score (AGS) for different aging subtypes in patient samples from the GSE135251 cohort. Specifically, we derived the negative AGS from Cluster 2 and the positive AGS from Clusters 6 and 8. The final AGS for each sample was determined by subtracting the negative AGS from the positive AGS. Differences in AGS between the two NMF clusters were assessed using the Wilcoxon test.

To assess differences in the immune microenvironment between the two NMF clusters, we employed the CIBERSORT algorithm to calculate infiltration scores for 22 immune cell types in G1 and G2 samples^58^. The Wilcoxon test was then used to evaluate differences in immune infiltration levels between the two clusters.

For Gene Set Enrichment Analysis (GSEA), we performed differential expression analysis between G1 and G2 groups using the R package DESeq2. Based on the fold-change values of individual genes, GSEA was conducted using the R package clusterProfiler. KEGG gene sets for enrichment analysis were obtained from the MSigDB database (https://www.gsea-msigdb.org/gsea/msigdb/index.jsp).

### Identification of biomarker genes

To select key features, we employed two machine learning algorithms: the Boruta^59^ algorithm and LASSO^60^ regression analysis. Using the R package Boruta, we performed 1000 iterations to assess the importance of each feature for selection. Additionally, LASSO regression analysis was conducted with the R package glmnet, applying 10-fold cross-validation and selecting feature genes based on the minimum lambda value (*λ* = 0.02).

### Construction and evaluation of the predictive nomogram

Using the R package rms, we constructed a nomogram to predict diagnostic probabilities. Calibration curves were plotted to evaluate the nomogram by comparing the predicted probabilities with the observed diagnostic probabilities.

### Screening of anti-liver aging drugs

To identify potential anti-liver aging drugs, we constructed a drug-small molecule-target network by integrating liver-specific aging genes, drug targets, and protein-protein interaction (PPI) networks. First, we identified 194 medicine food homology substances, along with their small molecules and target genes, by integrating data from the SwissTargetPrediction (http://swisstargetprediction.ch/), FooDB (https://foodb.ca/), and TCMSP (https://www.tcmsp-e.com/) databases, focusing on traditional Chinese and Mongolian medicine^61–63^. Additionally, liver-specific aging genes were used as seed nodes, and their PageRank values were calculated. A drug-target network was then established, with a restart probability of 0.7, and the Random Walk with Restart (RWR) algorithm was applied to score drugs in the network, facilitating the identification of potential candidates related to liver aging.

### Molecular docking analysis

To evaluate the binding affinity and interaction modes between candidate drugs and their target proteins, molecular docking analyses were performed using Discovery Studio (BIOVIA). The crystal structures of target proteins were retrieved from the Protein Data Bank (PDB), including AR (PDB ID: 1XOW), *CHRM3* (8EA0), *NCF1* (1KQ6), *CYP1A2* (2HI4), *IL1B* (1L2H), *PRKCB* (2I0E), *COL3A1* (4AE2), *CRP* (1B09), *CHRNA2* (5FJV), *NCAM2* (2XY1), and *FASN* (3TJM). For targets lacking experimentally resolved structures (*LUM*, *GPRC5B*, *EFEMP1*, and *DKK3*), predicted three-dimensional structures were obtained from the AlphaFold Protein Structure Database. The molecular structures of candidate compounds were downloaded from the PubChem database.

Prior to docking, protein structures were prepared using the Prepare Protein module by removing non-standard residues, adding polar hydrogen atoms, and optimizing protonation states. Ligand structures were processed and energy-minimized using the Prepare Ligands module. Binding sites were defined based on known active pockets or automatically detected using the From Current Selection option. Semi-flexible molecular docking was then performed using the CDOCKER algorithm, and the –CDOCKER ENERGY value was used to evaluate binding stability, with higher values indicating stronger predicted interactions.

### Reagents

Fetal bovine serum (FBS; C04001-500), high-glucose Dulbecco’s modified Eagle medium (DMEM; C3113-0500), and penicillin–streptomycin solution (C3420-0100) were purchased from ViVaCell. The Cell Counting Kit-8 (CCK-8; FC101-02) was obtained from TRNA. Alanine aminotransferase (ALT/GPT) and aspartate aminotransferase (AST/GOT) colorimetric assay kits (E-BC-K235-M) were purchased from Elabscience. Total cholesterol (A111-1-1) and triglyceride (A110-1-1) assay kits were obtained from Nanjing Jiancheng Bioengineering Institute. Oxidized low-density lipoprotein (ox-LDL; YB002) was purchased from Guangzhou Yiyuan Biotechnology, and the Oil Red O staining kit (G1262) was obtained from Beijing Solarbio.

### Animal and drug-containing serum preparation

Six male healthy SPF grade Wistar rats were used in this study. The rats were acclimatised for 3 days. The dose administered to the rats was calculated by converting the human equivalent dose (x mg/kg) using standard body surface area conversion formula (x mg/kg × 70 kg × 0.018/0.2 kg = 6.3 x mg/kg). *Fagopyrum dibotrys* (Jinqiaomai, abbreviated as JQM) extract was prepared by adding 7.5 g of crude buckwheat powder to 500 mL of distilled water, followed by reflux extraction for 1.5 h. The extract was filtered and concentrated to a final volume of 75 mL using rotary evaporation. Rats were administered the extract by gavage twice daily for seven consecutive days. At 1.5 h after the final administration, rats were anesthetized with urethane, and whole blood was collected from the abdominal aorta. Blood samples were centrifuged at 3000 rpm for 15 min to obtain serum. The collected serum was heat-inactivated at 56 °C for 30 min, sterilized by filtration through a 0.22 μm membrane, aliquoted, and stored at −80 °C until use.

In subsequent cell experiments, serum from rats administered *Fagopyrum dibotrys* was added to the culture medium at final concentrations of 2%, 4%, 6%, 8%, or 10%. To minimize variability caused by differences in total serum content, the final culture volume was fixed at 1.0 mL, and the total serum concentration was maintained at 10% across all experimental groups. This was achieved by supplementing JQM serum with fetal bovine serum (FBS) at different ratios. Corresponding control groups included cultures treated with JQM-free rat serum as well as a 10% FBS-only control. All sera used for in vitro experiments were heat-inactivated and sterile-filtered prior to use.

### Ethical reviews

At the end of the experiment, animals were anesthetized with 2.5% isoflurane. Euthanasia was performed by cervical dislocation and bilateral thoracotomy to ensure death. Prior to tissue collection, the absence of vital signs was confirmed, including cardiac arrest, unresponsiveness to toe-pinch reflex, and fixed, dilated pupils. All animal and human procedures were approved by the Institutional Review Committee of Harbin Medical University (IRB2020724) and followed the NIH Guide for the Care and Use of Laboratory Animals.

### HepG2 and RAW264.7 cell culture and treatment

HepG2 cells were cultured in Dulbecco’s modified Eagle’s medium (DMEM) supplemented with 10% heat-inactivated fetal bovine serum (FBS) and 1% penicillin–streptomycin, and maintained at 37 °C in a humidified incubator with 5% CO_2_. Cells in the logarithmic growth phase were used for all experiments. To establish an oxidative stress–induced hepatocyte injury model, HepG2 cells were treated with 150 μM H_2_O_2_ for 24 h, as previously described^64^. RAW264.7 mouse mononuclear phagocytes were cultured under the same conditions in DMEM containing 10% heat-inactivated FBS and 1% penicillin–streptomycin at 37 °C in a 5% CO_2_ incubator. Cells in the logarithmic growth phase were selected for subsequent experiments.

### CCK-8 assay

HepG2 cells grown in logarithmic phase were taken and inoculated in 96-well plates and randomly divided into blank group, model group and JQM containing serum group. The model group was cultured in H_2_O_2_ medium with a final concentration of 150 μM for 24 h. The JQM serum-containing group was pretreated in medium containing different concentrations of buckwheat-containing serum (2%, 4%, 6%, 8%, and 10%) for 24 h. The medium was replaced by H_2_O_2_ medium with a final concentration of 150 μM for 24 h. 10 μl of CCK-8 was added to each well and incubated in an incubator for 1 h. The OD value was measured at 450 nm on an enzyme marker and the cell viability was calculated.

### Measurement of AST and ALT levels

After HepG2 cells were processed, they were broken by ultrasound using saline (0.9% NaCl), and after centrifugation, the supernatants were taken and analysed for the levels of AST and ALT using the glutamate transaminase (ALT/GPT) colorimetric test kit and the aspartate transaminase (AST/GOT) colourimetric kit, according to the manufacturers’ instructions.

### Establishment of the foam cell model and Oil Red O staining

To induce foam cell formation, RAW264.7 cells were treated with 50 μg/mL oxidized low-density lipoprotein (ox-LDL) for 24 h. Following stimulation, cells were washed twice with phosphate-buffered saline (PBS) and fixed with fixative for 30 min at room temperature. After fixation, cells were rinsed twice with distilled water and briefly immersed in 60% isopropanol for 20–30 s to facilitate lipid staining. Freshly prepared Oil Red O working solution was then added, and cells were incubated in the dark for 1 h. Excess stain was removed by rinsing with 60% isopropanol for 10–20 s until the background became clear, followed by 2–5 washes with distilled water. Cell nuclei were counterstained with Mayer’s hematoxylin for 1–2 min and subsequently blued using Oil Red O buffer for 1 min. Finally, cells were maintained in distilled water and examined and imaged under a light microscope.

### Determination of Total Cholesterol (TC), Triglyceride (TG), and inflammatory and fibrosis-related markers

Cell suspensions were collected and centrifuged to obtain cell pellets, which were subsequently homogenized by sonication. Total cholesterol (TC) and triglyceride (TG) levels were measured according to the manufacturer’s instructions. Briefly, distilled water, calibrators, samples, and working reagent were added to each well, followed by gentle mixing. After incubation at 37 °C for 10 min, absorbance was measured at 500 nm using a microplate reader.

In addition, the expression levels of pro-inflammatory cytokines, including TNF-α and IL-1β, and the fibrosis-related factor TGF-β were determined in parallel. Briefly, cell culture supernatants or lysates were collected, and the concentrations of these markers were quantified using commercially available enzyme-linked immunosorbent assay (ELISA) kits according to the manufacturers’ protocols. Absorbance was measured using a microplate reader, and concentrations were calculated based on standard curves.

### Statistical analysis

All statistical analyses were conducted using R software (version 4.2.1). The Wilcoxon rank-sum test was used to compare non-normally distributed continuous variables between two groups, while Fisher’s exact test was applied to evaluate differences in categorical variables. All tests were two-tailed, and a *p*-value below 0.05 was considered indicative of statistical significance. To control for false discovery rates in multiple comparisons, *p*-values were adjusted using the Benjamini-Hochberg method.

## Supplementary information

Additional file 1-Supplementary Figures and Figure Captions

Supplementary Data 1

Supplementary Data 2

Supplementary Data 3

## Data Availability

All data generated or analyzed during this study are included in this published article. Additional raw data are available from the corresponding author upon reasonable request.

## Code availability

The code used for data processing and analysis in this study is available from the corresponding author upon reasonable request.
