# Supplementary material for: Integrated transcriptomic analysis identifies liver aging-driven fibrosis signatures and reveals therapeutic strategies based on medicine-food homology
Source: NPJ Sci Food. 2026 May 26;10:252. doi: 10.1038/s41538-026-00904-6 (PMC13486760; doi:10.1038/s41538-026-00904-6)
Supplement: Supplementary file 1 [file 41538_2026_904_MOESM1_ESM.pdf]

Fig. S1

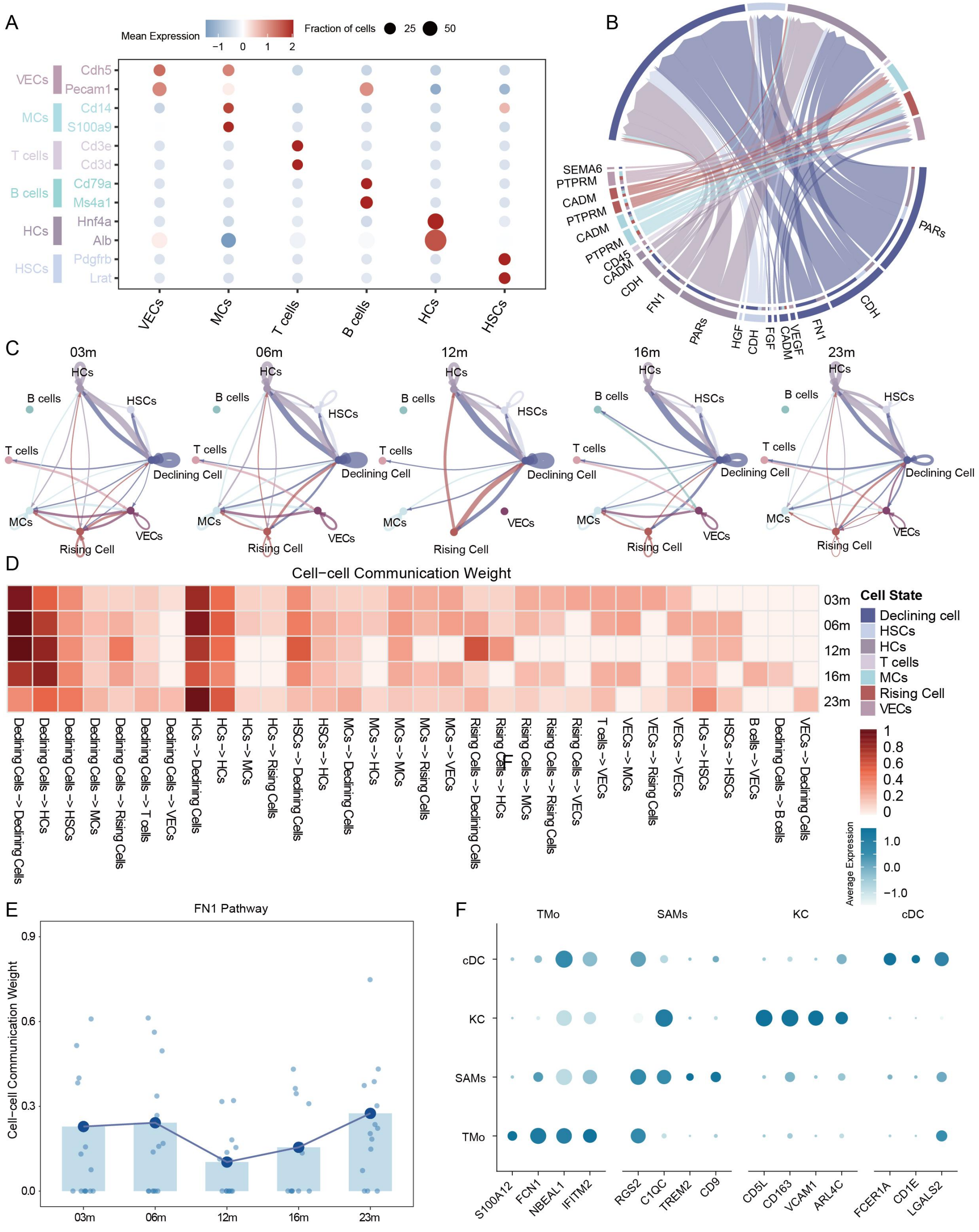

A
Mean Expression
Fraction of cells 25 50
VECs
MCs
T cells
B cells
HCs
HSCs
Cdh5
Pecam1
Cd14
S100a9
Cd3e
Cd3d
Cd79a
Ms4a1
Hnf4a
Alb
Pdgfrb
Lrat
B
SEMA6
PTPRM
CADM
CD45
CDH
FN1
PARs
HGF
FGF
VEGF
C
03m
06m
12m
16m
23m
Declining Cell
Rising Cell
D
Cell−cell Communication Weight
Cell State
Declining cell
HSCs
HCs
T cells
MCs
Rising Cell
VECs
Average Expression
E
FN1 Pathway
Cell−cell Communication Weight
F
TMo
SAMs
KC
cDC
S100A12
FCN1
NBEAL1
IFITM2
RGS2
C1QC
TREM2
CD9
CD5L
CD163
VCAM1
ARL4C
FCER1A
CD1E
LGALS2

**Fig. S1. Identification of cell types and characterization of cellular communication.**

**A.** Dot plot showing the average expression of canonical marker genes used to identify six major cell types in dataset GSE247719. **B.** Circos plot depicting the overall interaction strength between different cell types. **C.** Cell communication networks illustrating the interaction strength between Rising/Declining cells and other major cell types across five age points (3, 6, 12, 16, and 23 months). **D.** Heatmap showing the inferred cell－cell interaction strength across five time points, as determined by CellChat. **E.** Scatter plot illustrating the total inferred cell-cell interaction strength of the fibrosis-associated FN1 signaling pathway across five time points. **F.** Dot plot displaying the average expression of marker genes used to classify four mononuclear phagocyte (MP) subtypes in dataset GSE136103.

# Fig. S2

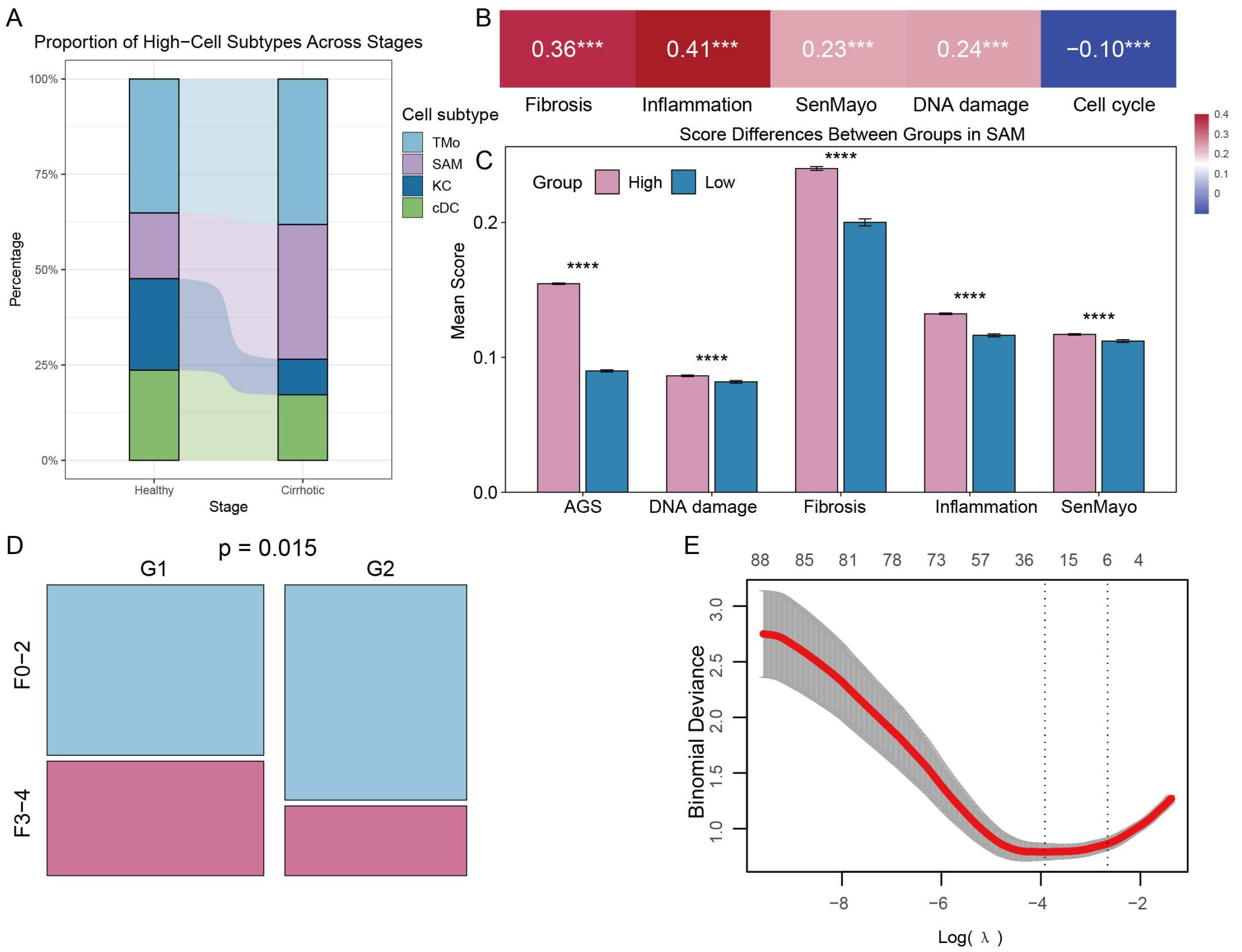


**A.** Bar graph comparing the relative proportions of the four MP subtypes among high AGS cells in healthy versus cirrhotic liver samples. **B.** Heatmap showing the correlations between AGS scores and gene signatures related to fibrosis, inflammation, classical aging, DNA damage, and the cell cycle. **C.** Bar plot illustrating the correlations between AGS scores and these gene signature scores across different AGS groups in SAM cells. **D.** Stacked bar plot showing the distribution of fibrosis stages (F0 – 2 and F3 – 4) between the G1 and G2 subtypes, with statistical significance indicated. **E.** Cross-validation error plot for parameter tuning in the Lasso regression model.

**Fig. S3**

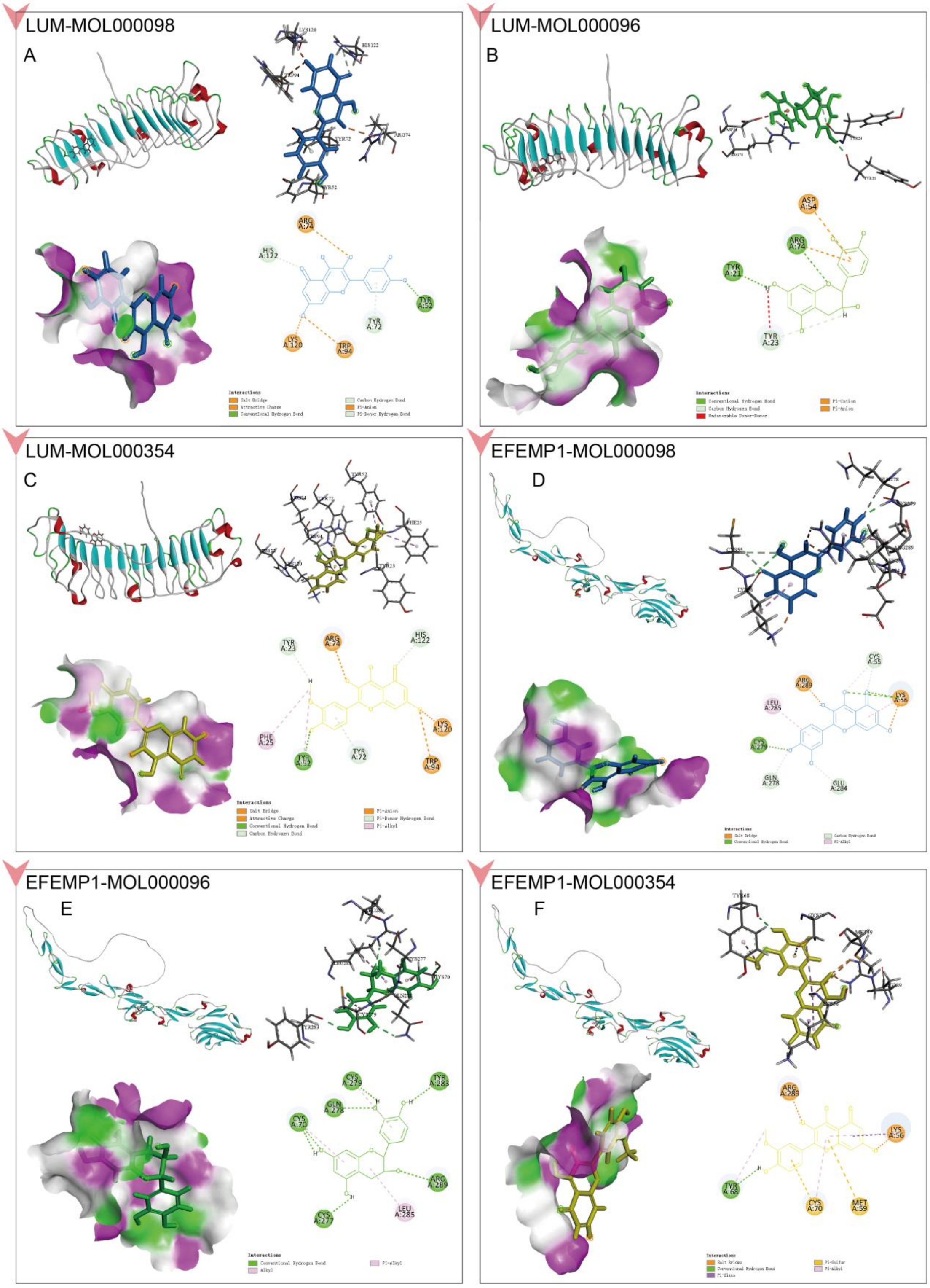
LUM-MOL000098
A
LUM-MOL000096
B
LUM-MOL000354
C
EFEMP1-MOL000098
D
EFEMP1-MOL000096
E
EFEMP1-MOL000354
F

**Fig. S3. Molecular docking visualization of candidate compounds with fibrosis-related targets.**

**A-C.** Predicted binding conformation, binding pocket structure (highlighting hydrogen bond donors/acceptors), 3D interaction diagram, and 2D interaction map of compounds MOL000098 (A), MOL000096 (B), and MOL000354 (C) with the target protein LUM. **D-F.** Corresponding molecular docking visualizations of the same compounds MOL000098 (D), MOL000096 (E), and MOL000354 (F) with the target protein EFEMP1.

# Fig. S4

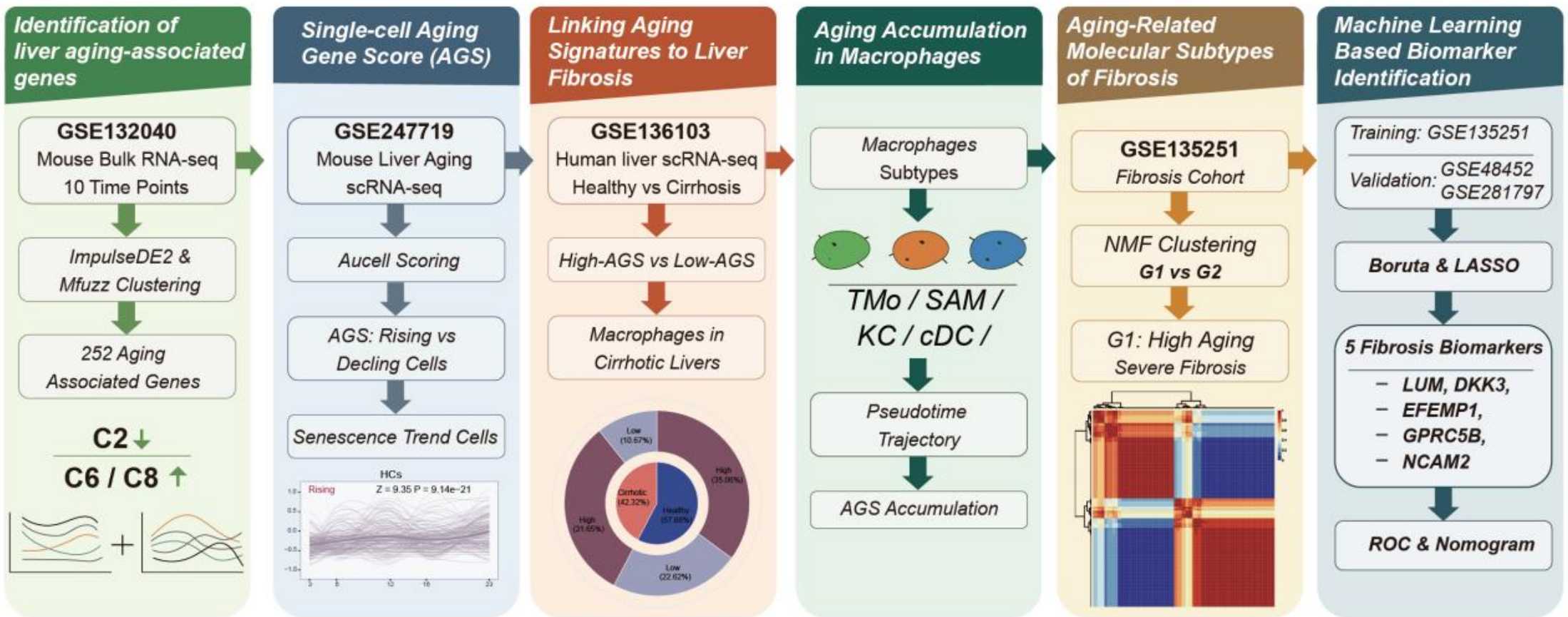


**Fig. S4. Overview of the data integration and analytical pipeline used in this study.**

The schematic illustrates the multi-dataset integration strategy and key analytical steps. Bulk RNA-seq time-course data (GSE132040) were leveraged to identify liver-specific aging–associated genes with dynamic expression trends. Single-cell RNA-seq datasets (GSE247719 and GSE136103) were used to construct a single-cell senescence landscape and to perform trajectory analysis of macrophage subpopulations. Clinical fibrosis cohorts (GSE135251, GSE48452, and GSE281797) enabled the evaluation of aging signatures in relation to fibrosis progression, biomarker selection, and nomogram development. Integrated analyses across these layers connected transcriptional aging dynamics, cell-type–specific senescence states, and fibrotic pathology, and further facilitated the systematic screening of medicine–food homology candidates with potential therapeutic relevance.

**Supplementary Data 1 | Drug prioritization scores calculated using a random walk with restart (RWR) algorithm.**

Drug rankings were generated by applying a random walk with restart (RWR)–based drug screening model, using fibrosis-associated marker genes as input features.

**Supplementary Data 2 | Molecular docking scores between fibrosis-associated marker proteins and small-molecule components of Jinqiaomai.**

This Excel file provides molecular docking scores for interactions between fibrosis-associated marker proteins and three small-molecule compounds identified in Jinqiaomai.

**Supplementary Data 3 | Molecular dynamics–based interaction scores between fibrosis-associated marker proteins and Jinqiaomai-derived small molecules.**

This Excel file reports molecular dynamics simulation–derived scores for interactions between fibrosis-associated marker proteins and three small-molecule compounds identified in Jinqiaomai.
